# Functional expression and characterization of the envelope glycoprotein E1E2 heterodimer of hepatitis C virus

**DOI:** 10.1371/journal.ppat.1007759

**Published:** 2019-05-22

**Authors:** Longxing Cao, Bowen Yu, Dandan Kong, Qian Cong, Tao Yu, Zibo Chen, Zhenzheng Hu, Haishuang Chang, Jin Zhong, David Baker, Yongning He

**Affiliations:** 1 State Key Laboratory of Molecular Biology, National Center for Protein Science Shanghai, Shanghai Science Research Center, CAS Center for Excellence in Molecular Cell Science, Shanghai Institute of Biochemistry and Cell Biology, Chinese Academy of Sciences; University of Chinese Academy of Sciences, Shanghai, China; 2 Department of Biochemistry, University of Washington, Seattle, Washington, United States of America; 3 Institute for Protein Design, University of Washington, Seattle, Washington, United States of America; 4 CAS Key Laboratory of Molecular Virology and Immunology, Unit of Viral Hepatitis, Institut Pasteur of Shanghai, Chinese Academy of Sciences, Shanghai, China; 5 Howard Hughes Medical Institute, University of Washington, Seattle, Washington, United States of America; Purdue University, UNITED STATES

## Abstract

Hepatitis C virus (HCV) is a member of *Hepacivirus* and belongs to the family of *Flaviviridae*. HCV infects millions of people worldwide and may lead to cirrhosis and hepatocellular carcinoma. HCV envelope proteins, E1 and E2, play critical roles in viral cell entry and act as major epitopes for neutralizing antibodies. However, unlike other known flaviviruses, it has been challenging to study HCV envelope proteins E1E2 in the past decades as the *in vitro* expressed E1E2 heterodimers are usually of poor quality, making the structural and functional characterization difficult. Here we express the ectodomains of HCV E1E2 heterodimer with either an Fc-tag or a *de novo* designed heterodimeric tag and are able to isolate soluble E1E2 heterodimer suitable for functional and structural studies. Then we characterize the E1E2 heterodimer by electron microscopy and model the structure by the coevolution based modeling strategy with Rosetta, revealing the potential interactions between E1 and E2. Moreover, the E1E2 heterodimer is applied to examine the interactions with the known HCV receptors, neutralizing antibodies as well as the inhibition of HCV infection, confirming the functionality of the E1E2 heterodimer and the binding profiles of E1E2 with the cellular receptors. Therefore, the expressed E1E2 heterodimer would be a valuable target for both viral studies and vaccination against HCV.

## Author summary

Hepatitis C virus (HCV) is an enveloped virus that infects millions of people worldwide and may lead to cirrhosis and hepatocellular carcinoma. HCV has two envelope proteins, E1 and E2, which form heterodimers on viral surface and are critical for HCV cell entry. However, current studies of HCV E1E2 are often limited by the poor quality of the *in vitro* expressed E1E2 heterodimers. Here we express the ectodomains of HCV E1E2 with different tags, and are able to isolate soluble E1E2 ectodomains suitable for structural and functional studies. Then we generate the 3D reconstruction of E1E2 heterodimer by electron microscopy and also model the E1E2 structure by the coevolution based strategy with Rosetta, showing the potential interactions between E1 and E2. Moreover, the E1E2 heterodimer is applied to examine the interactions with the HCV cellular receptors, neutralizing antibodies as well as the inhibition of HCV infection. These results suggest that the expressed E1E2 heterodimer would be a promising target for both viral studies and vaccination against HCV.

## Introduction

Hepatitis C virus (HCV) is an enveloped positive-stranded RNA virus that belongs to the genus *Hepacivirus* in the family of *Flaviviridae* [1, 2]. Its genome consists of a single open reading frame encoding a protein product, which is cleaved by cellular and viral proteases into ten smaller proteins, including three structural proteins, namely core protein, E1 and E2, and seven nonstructural proteins [3]. HCV causes both acute and chronic infections, and the chronic infection may lead to liver diseases such as cirrhosis, hepatocellular carcinoma and liver failure [4]. According to the statistics from WHO, approximately 71 million people have chronic HCV infection globally and nearly 400,000 people die each year from hepatitis C, mostly through cirrhosis and hepatocellular carcinoma. Current antiviral medicines against HCV show high cure rates (>95%), but the high cost, side effects, viral resistance and the potential of reinfection [5–7] are limiting the antiviral effects. Up to date, no vaccine is available for HCV, largely due to its high polymorphism in genotypes and morphologies. The lack of structural information also hampers the development of HCV vaccines.

HCV has two envelope glycoproteins, E1 and E2, which mediate the cell entry through the interactions with host cell receptors and are promising targets for vaccine development. A large number of studies have shown that several cell surface receptors are involved in HCV cell entry. Among them, glycoprotein E2 has been reported to interact directly with tetraspanin/CD81 [8–11], scavenger receptor class B member 1 (SR-B1) [12, 13] and very low density lipoprotein receptor (VLDLR) [14]. Glycoprotein E1 is suggested to be responsible for the fusion between viral and cellular endosomal membranes during HCV entry process and it might also interact with the apolipoprotein E (ApoE) [15, 16]. In addition, several other receptors have also been reported to be important for HCV cell entry, for example, claudin-1 (CLDN1) [17], occludin (OCLN) [18], and NCP1L1 [19]. However, the exact roles of these receptors in viral entry are not fully understood [20].

Both E1 and E2 of HCV are type I transmembrane proteins containing an N-terminal ectodomain (160 residues for E1 and 330 residues for E2) and a well-conserved C-terminal transmembrane domain of about 30 amino acids [21]. E1 and E2 form heterodimers on viral surface, which is important for the maturation as well as the infectivity of viral particles, and their transmembrane domains might be involved in the heterodimerization process [20, 22]. E1 and E2 ectodomains have five and eleven potential glycosylation sites, respectively, and these carbohydrates might be important for the stability and antigenicity of HCV particles [23]. Moreover, E1 and E2 ectodomains also contain eight and eighteen cysteines, respectively, which can form both intra- and inter- molecular disulfide bonds that may affect the host receptor interactions [20, 24]. Glycosylation and disulfide bonds might be critical for the folding and maturation of E1 and E2 as overexpression of these proteins often results in mis-folded disulfide bond-linked aggregates [25, 26]. Up to date, only partial structural information of E1E2 heterodimer is available. The crystal structure of an N-terminal fragment of E1 ectodomain shows a covalently linked domain-swapped homodimer [27]. The core of E2 has been solved in complex with antibodies [28–30], where E2 adopts a central immunoglobin-like fold formed by β-sheets surrounded by short α-helices dispersed in loops. However, both E1 and E2 used for structural studies are truncated proteins, part of the ectodomains are missing in the solved structures [31, 32]. Meanwhile, several groups have been trying to co-express E1E2 heterodimer to ensure the correct folding of the intact proteins as the folding and maturation of E1 and E2 may depend on each other [33–36]. Unfortunately, co-expression of E1E2 heterodimer often leads to poor quality samples and the structural characterization of the intact E1E2 heterodimer has not been successful.

Here we expressed the ectodomains of HCV E1E2 heterodimer fused with either an Fc-tag or a *de novo* designed heterodimeric tag and characterized the structure of E1E2 heterodimer with both electron microscopic reconstruction and the coevolution-guided modeling using Rosetta. Moreover, we examined the interactions of the E1E2 heterodimer with the known HCV receptors and neutralizing antibodies and the inhibition of HCV infection by the heterodimer, suggesting that the expressed E1E2 heterodimer is functional and could be a valuable target for further structural studies and vaccine development against HCV.

## Results

### Expression of E1E2 as an Fc-tagged heterodimer in insect cells

**Fig 1:**
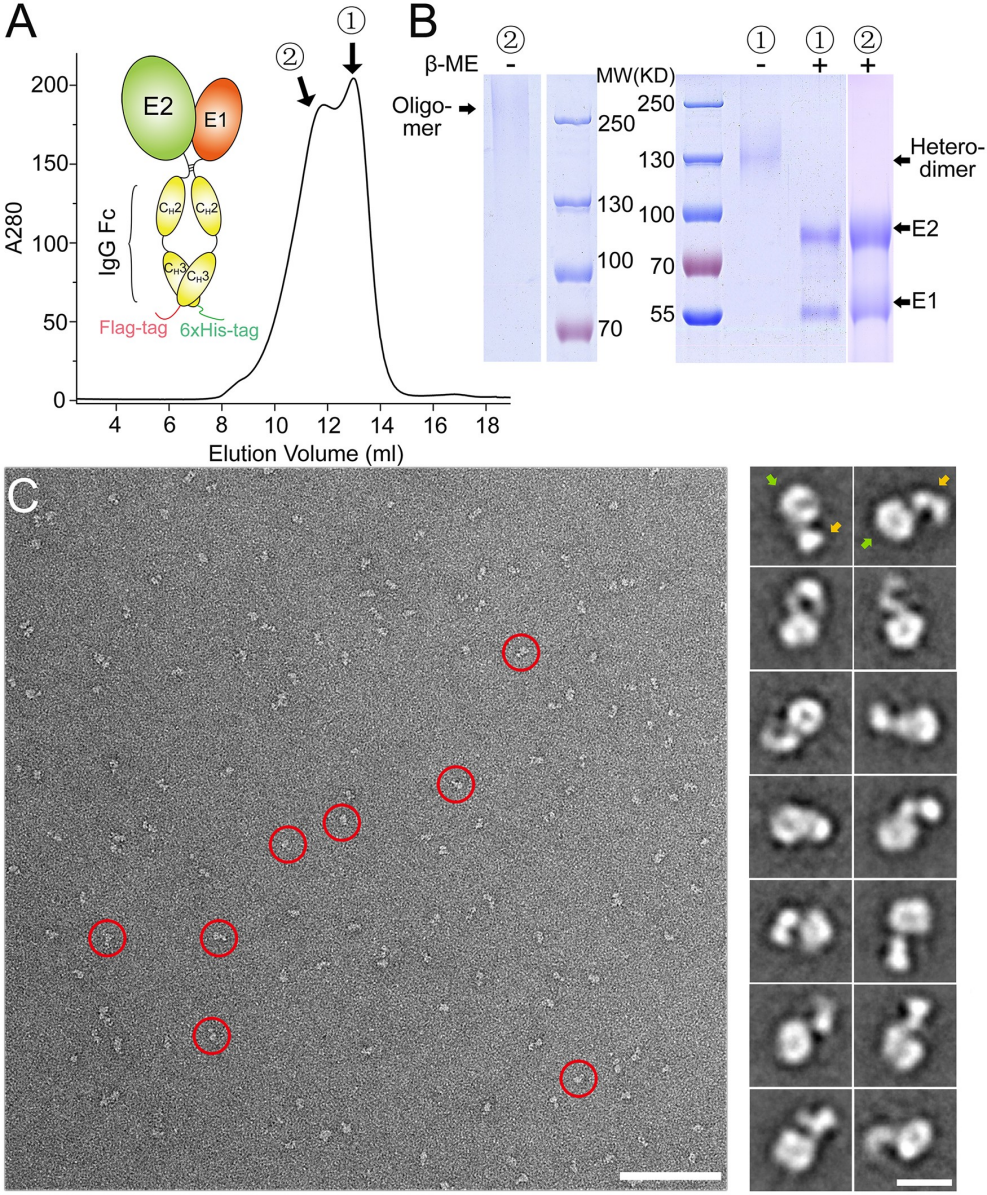
Expression of HCV E1E2 as an Fc-tagged heterodimer in insect cells. (A) Schematic representation of the Fc-tagged HCV E1E2 heterodimer and the SEC profile of the purified E1E2-Fc. The heterodimeric and oligomeric peaks of E1E2-Fc are labeled as 1 and 2, respectively. (B) SDS-PAGE of the purified E1E2-Fc under reducing and non-reducing conditions for the two peaks shown in (A). (C) A negative staining EM image showing the heterodimeric E1E2-Fc particles (left; red circles; bar, 100 nm). The representative 2D averaging classes are also shown (right; bar, 10 nm). The head and the tail regions are indicated by green and orange arrows, respectively.

In order to obtain E1E2 heterodimer suitable for functional and structural studies, we first tried to co-express E1 and E2 ectodomains (from HCV strain H1b) in insect cells by replacing the transmembrane domains of E1 and E2 with a Flag-tag and a 6xHis-tag, respectively. But almost no expression of E1 and low expression of E2 were detected. This is not surprising as the folding of E1 and E2 may depend on each other, and the transmembrane domains of E1 and E2 have been shown to be important for the formation of E1E2 heterodimer [22]. Therefore, we designed a construct using an IgG Fc fragment to substitute the transmembrane domains of E1 and E2 (Fig 1A), where the IgG Fc fragments would dimerize and may facilitate the formation of E1E2 heterodimer. To isolate the E1E2 heterodimer, a Flag-tag at the C-terminus of E1-Fc and a 6xHis-tag at the C-terminus of E2-Fc were added for affinity chromatography (Fig 1A). The construct was expressed as secreted soluble forms in insect cells and the purified E1E2 heterodimers were obtained after two consecutive affinity purification steps with Ni-NTA resin and anti-Flag M2 resin. The size-exclusion chromatography (SEC) showed that the E1E2 proteins contained both heterodimers and oligomers, which correspond to the two peaks in the chromatogram (Fig 1A). Proteins from both peaks were loaded onto SDS-PAGE under reducing and non-reducing conditions (Fig 1B). The peak that corresponds to the heterodimers showed a single band with the molecular weight equal to E1E2-Fc under non-reducing conditions, while E1 and E2 were separated under reducing conditions, which is not unexpected as there are disulfide bonds in Fc tag. Similarly, the peak that corresponds to the oligomer also showed a smeared band with high molecular weights under non-reducing conditions (Fig 1B), suggesting they might be disulfide bond-linked oligomers, which has been reported before [20, 24, 37–40]. The E1E2 heterodimer was collected and showed mono-dispersed particles by negative stain EM imaging (Fig 1C). The two-dimensional (2D) class averages of the boxed particles showed that E1E2-Fc contained a head and a tail region. The head region should correspond to the E1E2 heterodimer and the tail region is formed by the Fc homodimer (Fig 1C). The E1E2 oligomers were also collected for negative stain EM, and the images showed larger particles with blurry 2D class averages, implying that the oligomers might be heterogeneous (S3A Fig).

During the expression of E1E2 with Fc tag, E2-Fc homodimer was also found as expected, however, no E1-Fc homodimer was detected in supernatant, suggesting that E2 might be required for the secretion of E1. To validate this result, both E1-Fc and E2-Fc were expressed individually using the similar expression system in insect cells, and indeed, E2-Fc can be found in supernatant (S1A Fig), whereas E1-Fc can only be detected in cell pellets (S1C Fig), suggesting that E1-Fc alone may not fold properly and are retained intracellularly. The mono-dispersed particles of E2-Fc homodimer could be seen on the negatively stained EM images and the 2D class averages also showed that E2-Fc had two regions corresponding to the E2 homodimer and the Fc tail, respectively (S1B Fig). And the E2 homodimer revealed different features from the E1E2 heterodimer in the 2D averaged images, which is expected and confirms the formation of E1E2 heterodimer with Fc tag.

### Expression of E1E2 heterodimer with a *de novo* designed heterodimeric tag in insect cells

**Fig 2:**
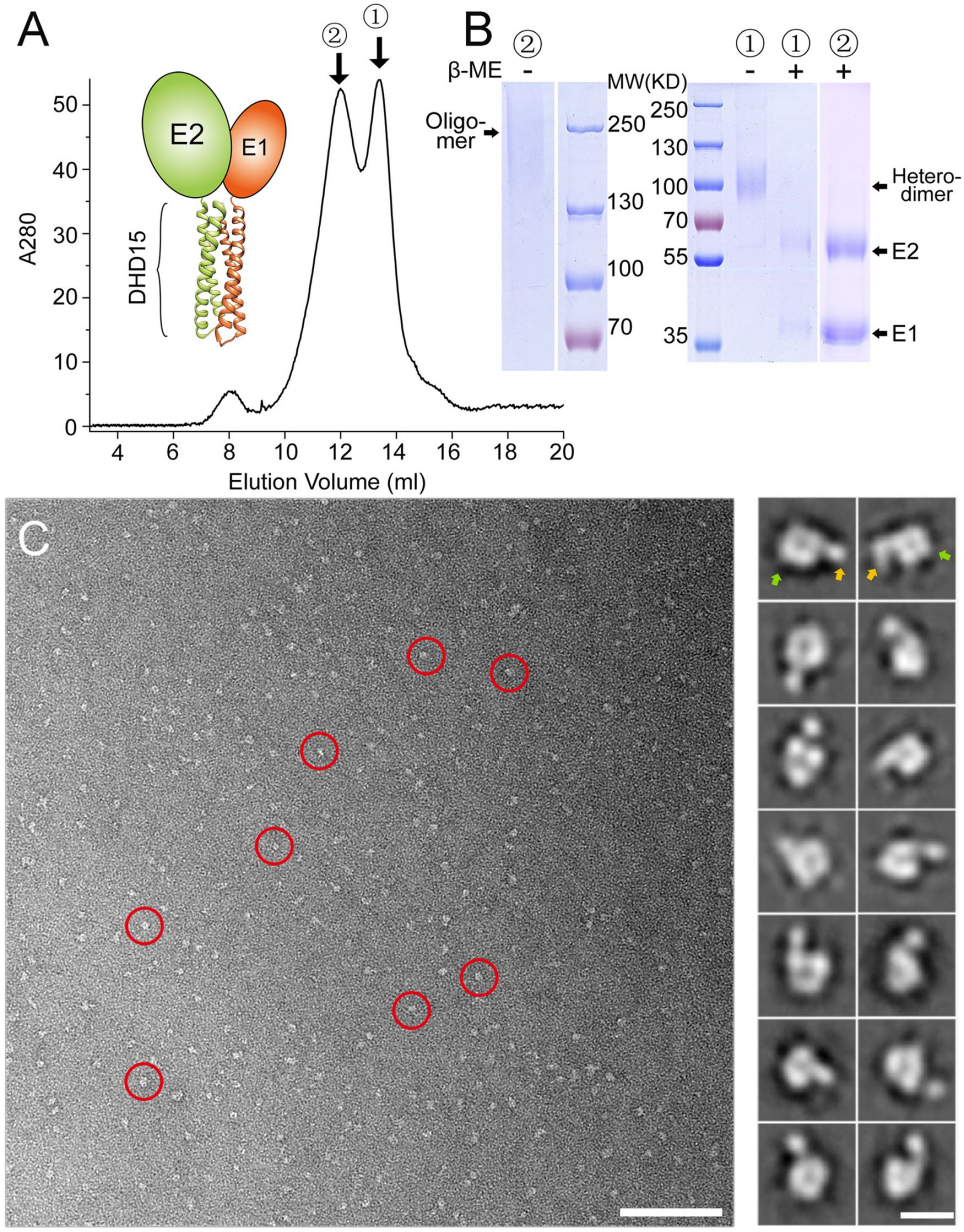
Expression of HCV E1E2 using a *de novo* designed heterodimeric tag DHD15 in insect cells. (A) Schematic representation of the HCV E1E2 heterodimer with a *de novo* designed tag DHD15 and the SEC profile of the purified E1E2-DHD15 expressed in insect cells. The heterodimeric and oligomeric peaks of E1E2-DHD15 are labeled as 1 and 2, respectively. (B) SDS-PAGE of the purified E1E2-DHD15 under reducing and non-reducing conditions for the two peaks shown in (A). (C) A negative staining EM image showing the heterodimeric E1E2-DHD15 particles (left; red circles; bar, 100 nm). The representative 2D averaging classes are also shown (right; bar, 10 nm). The head and the tail regions are indicated by green and orange arrows, respectively.

To mimic the native folding and maturation of E1E2, we used a pair of *de novo* designed helical hairpins (DHD15, PDB entry: 6DMA) to replace the transmembrane domains of E1 and E2 (Fig 2A). The designed helical hairpins only form heterodimers specifically [41], therefore could maximize the yield of E1E2 heterodimer. In addition, the N-terminus of each helical hairpin locates close to each other, allowing the direct fusion of E1 and E2. Furthermore, the *de novo* designed hairpin heterodimer is thermal stable, which may facilitate the folding and maturation of E1E2 heterodimer.

The construct of E1E2-DHD15 was expressed in insect cells and purified using both Flag-tag and His-tag followed by SEC as described above. Similarly, the SEC profile showed two peaks for E1E2-DHD15 (Fig 2A), which corresponded to E1E2 heterodimer and oligomer, respectively. The E1E2-DHD15 heterodimer was loaded onto SDS-PAGE under both reducing and non-reducing conditions (Fig 2B). The non-reducing SDS-PAGE showed a single band with the molecular weight equal to E1E2-DHD15, while the bands of both E1 and E2 are detected under reducing conditions. Since there is no disulfide bond in the DHD15 heterodimeric tag, the results suggest that disulfide bonds might be formed between E1 and E2 in the expressed heterodimers. Meanwhile, the fractions from the oligomer peak were also loaded onto SDS-PAGE under both reducing and non-reducing conditions (Fig 2B), the non-reducing SDS-PAGE showed a smeared band with high molecular weights, while the bands of E1 and E2 are separated under reducing conditions, confirming the formation of disulfide bond-linked oligomers during expression, which is consistent with the data reported before [24]. Moreover, fractions from both peaks were negatively stained and observed under EM. The images of the heterodimer showed uniform particles and the 2D averaged images revealed a doughnut-shaped head and a tail, which correspond to the E1E2 heterodimer and the DHD15 tag, respectively (Fig 2C). By contrast, the EM images of the E1E2-DHD15 oligomer showed larger heterogeneous particles (S3B Fig), consistent with the results of SEC and SDS-PAGE.

### Expression of E1E2 heterodimer in mammalian cells

**Fig 3:**
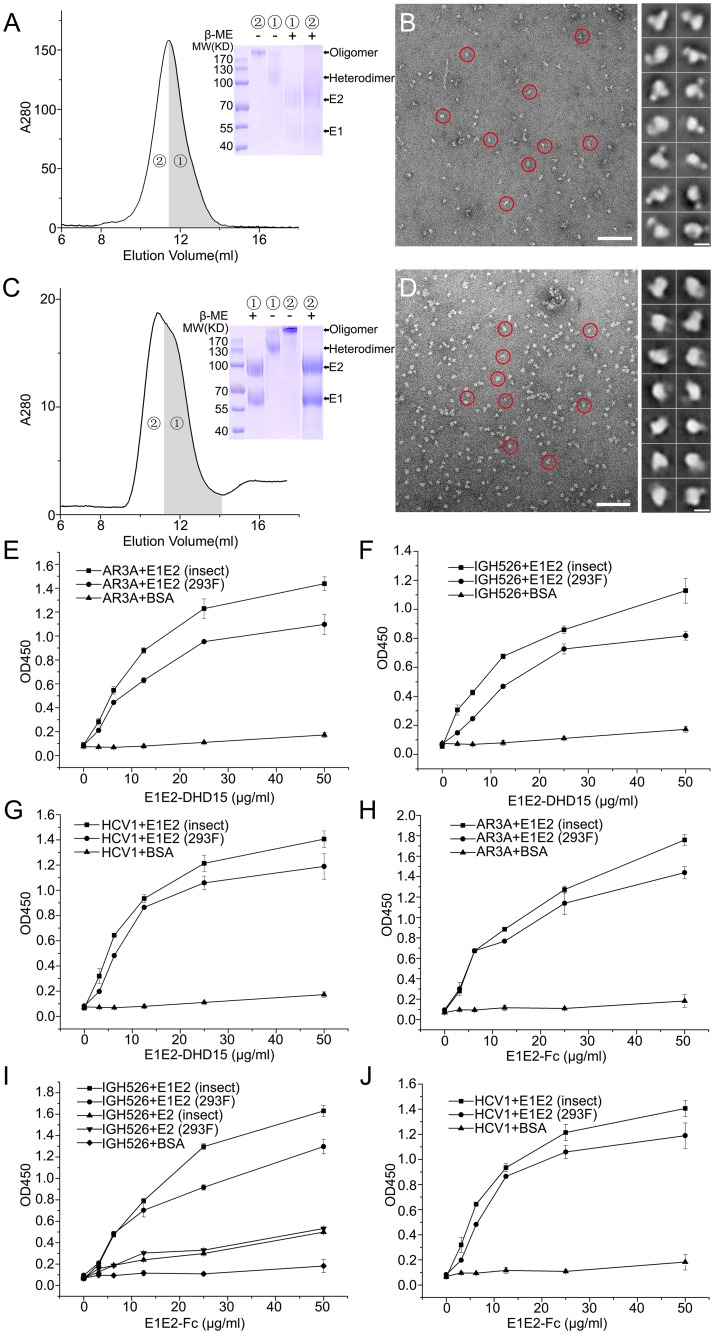
Expression of HCV E1E2 heterodimers in mammalian cells and the interactions of E1E2-DHD15 or E1E2-Fc heterodimer with neutralizing antibodies. (A) The SEC profile and the SDS-PAGE under reducing and non-reducing conditions of the purified E1E2-DHD15 expressed in HEK293 cells. (B) A negative staining EM image of the E1E2-DHD15 particles (left; red circles; bar, 100 nm) and the representative 2D averaging classes (right; bar, 10 nm). (C) The SEC profile and the SDS-PAGE under reducing and non-reducing conditions of the purified E1E2-Fc expressed in HEK293 cells. (D) A negative staining EM image of the E1E2-Fc particles (left; red circles; bar, 100 nm) and the representative 2D averaging classes (right; bar, 10 nm). (E)-(J) ELISA data show the binding of the insect or mammalian cell expressed E1E2-DHD15 or E1E2-Fc with neutralizing antibodies AR3A, IGH526 and HCV1, respectively. The ELISA data shown in (E)-(J) are representative of three repeated experiments and presented as mean ± SD.

Glycosylation has been shown to be important for the function of E1E2 [23]. Considering the different glycosylation patterns generated by insect and mammalian cells, we expressed both DHD15-tagged and Fc-tagged E1E2 heterodimer in mammalian cells. Both E1E2-DHD15 and E1E2-Fc expressed in HEK293 cells showed smeared bands with higher molecular weights on SDS-PAGE (Fig 3A and 3C), which is expected as mammalian cells usually produce larger and nonuniform glycosylation patterns. The SEC peak of E1E2-DHD15 also contained two species, E1E2 heterodimer and oligomer (Fig 3A), and both of them showed single bands under non-reducing conditions, whereas E1 and E2 were separated under reducing conditions, indicating the formation of disulfide-bond linked heterodimer or oligomer, which is similar to the proteins expressed in insect cells described above. The E1E2-Fc expression in HEK293 cells behaved similarly as E1E2-DHD15 (Fig 3C). These results suggest that the disulfide-bond linked E1E2 heterodimer and oligomer are formed independent of the expression systems, which is in agreement with data from other groups [20, 24, 37–40]. In addition, the negatively stained EM images also showed mono-dispersed particles for both samples (Fig 3B and 3D), however, the 2D averaged images did not show clear features as the insect cell expressed proteins, probably due to the heterogeneity resulting from the larger and nonuniform glycosylation of the samples.

Similar to the insect cell expression system, E2-Fc alone could be expressed and secreted properly by mammalian cells (S2A Fig), but no E1-Fc could be detected in supernatant if expressed by itself (S2C Fig). The negatively stained EM images showed that the mammalian cell expressed E2-Fc were also mono-dispersed, and the head and the tail regions can be seen in the 2D averaged images (S2B Fig). In parallel, we also expressed the E1E2 heterodimer from a different HCV strain, genotype 1a H77, and the similar results were obtained (S2D Fig), suggesting that the co-expression system described above could be applied to other HCV strains to obtain soluble E1E2 heterodimers.

In the meantime, we also treated E1E2 heterodimer with Endoglycosidase H (Endo H) as it has been shown that the virion-associated mature HCV glycoproteins are resistant to Endo H treatment [24, 39]. Indeed, the E1E2 proteins expressed in HEK293 cells were resistant to Endo H treatment (S6B Fig), whereas the insect cell expressed E1E2 proteins could be slightly deglycosylated by Endo H (S6A Fig). The negatively stained EM images were also collected for the Endo H treated E1E2-DHD15 expressed in insect cells, and the images showed well-dispersed particles sharing similar features with the untreated proteins (S6C Fig).

### Interactions of E1E2 proteins with HCV neutralizing antibodies

To validate the folding of the expressed E1E2 proteins, we tested the interactions of the Fc- and DHD15-tagged E1E2 heterodimers with the known HCV neutralizing antibodies. AR3A is an E2-specific antibody recognizing a discontinuous epitope on E2 and has been shown to be able to block the binding of E1E2 to CD81 [42, 43]. The ELISA data showed that AR3A could bind to the E1E2 heterodimers expressed in both insect and mammalian cells (Fig 3E and 3H). In parallel, the Fab fragments of neutralizing antibody IGH526, which recognizes a conformational epitope on E1 and may also have minimal binding activity to E2 [44], and antibody HCV1, which binds to a β-hairpin motif on E2 [45], were expressed and purified from HEK293 cells (S9 Fig). Similarly, the binding data showed that both IGH526 and HCV1 could bind to the E1E2 heterodimer expressed in both insect and HEK293 cells (Fig 3). In particular, IGH526 showed much higher binding activity to E1E2 heterodimer than E2 homodimer (Fig 3I), consistent with the reported data for this antibody [44]. Therefore, these results suggest that the E1E2 proteins expressed by our strategies have the similar epitopes as the E1E2 on viral surface. Furthermore, we also tested the binding of the oligomeric fractions of E1E2 with the neutralizing antibodies described above, and the data showed that E1E2 oligomers could bind to the neutralizing antibodies as well (S7 Fig), suggesting that the oligomers of E1E2 also contain correct epitopes.

### Three-dimensional EM reconstruction of E1E2 heterodimer

**Fig 4:**
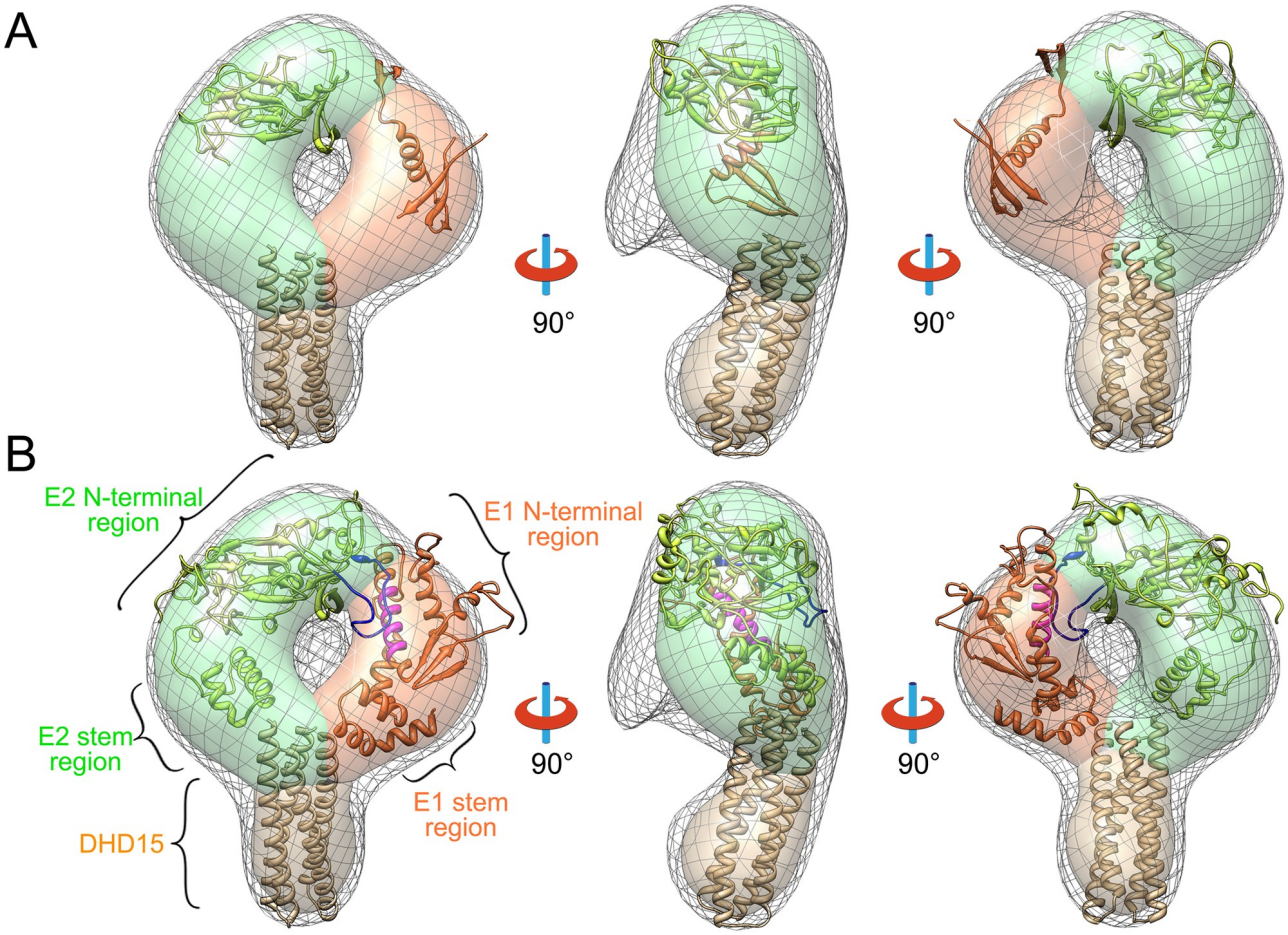
Three-dimensional EM reconstruction and the structural model of HCV E1E2 heterodimer. (A) Three views of the 3D reconstruction of E1E2-DHD15 (low-density contour: gray mesh; high-density contour: surface). The solved structures of E2 (PDB entry: 4MWF, green), E1 (PDB entry: 4UOI, orange) and DHD15 (brown) are put into the EM density showing that a large portion of E1 and E2 are missing in the known crystal structures. The densities corresponding to E2, E1 and DHD15 are colored in green, orange and brown in the high-density contour, respectively. (B) Three views of the coevolution based E1E2 structural model fitted into the EM reconstruction. The hyper variable region 2 (HVR2, blue) of E2 and the putative fusion peptide (magenta) of E1 are shown in the structural model.

To investigate the structure of E1E2, we applied 3D electron microscopy reconstruction to the E1E2 heterodimers expressed in insect cells as they showed mono-dispersed homogenous particles on negative staining EM images. The 2D averaged images showed that the E1E2 region of the E1E2-Fc heterodimer adopted a doughnut-like conformation (Fig 1C). But the 3D reconstruction based on the 2D images was not successful as the Fc portion, which occupies roughly one third of the total volume, was rather flexible relative to the E1E2 portion, thus making the particle alignment difficult for 3D reconstruction. By contrast, DHD15 tag is smaller with relatively low flexibility, allowing us to reconstruct a 3D model of E1E2-DHD15 heterodimer based on the negatively stained EM images at ~27 Å resolution (Fig 4).

The EM reconstruction of E1E2-DHD15 showed a volume with a doughnut-like head and a tail (Fig 4). The *de novo* designed helical bundle DHD15 can be fitted into the tail volume reasonably well (Fig 4A). The head that corresponds to the E1E2 heterodimer can be roughly divided into two density blobs at the high-density contour level (Fig 4A), which might correspond to the ectodomains of E2 and E1, respectively. Docking of the known crystal structures of E1 and E2 into the EM volume is difficult as these structures are truncated and only occupy roughly 50% of total EM volume (Fig 4A) [27–29].

It has been reported that during HCV entry, viral particles are internalized and transported into endosomes where E1E2 may undergo conformational changes in response to acidic pH [46–48]. Therefore, we incubated E1E2-DHD15 in acidic buffer (pH 5.5) overnight and imaged under EM. The resulting 2D class averages and the 3D reconstruction reveal no obvious difference (S4 Fig), suggesting that the pH-induced conformational change of E1E2 may not be large enough to be visible at low resolution. Another possibility is that the E1E2 heterodimer requires post-attachment priming steps before it responds to low pH during viral entry [47], as in the case of pestiviruses [49].

### Coevolution-based structural modeling of E1E2 heterodimer

To further explore the structure of E1E2 heterodimer, we modeled the E1E2 heterodimer by combining the coevolution analysis with GREMLIN [50] and the molecular modeling with Rosetta [51, 52] in the context of the 3D EM model. According to the coevolution theory, the coevolving residues within or between proteins usually form spatial contacts, and such information would facilitate protein structure prediction by Rosetta [53–55].

**Fig 5:**
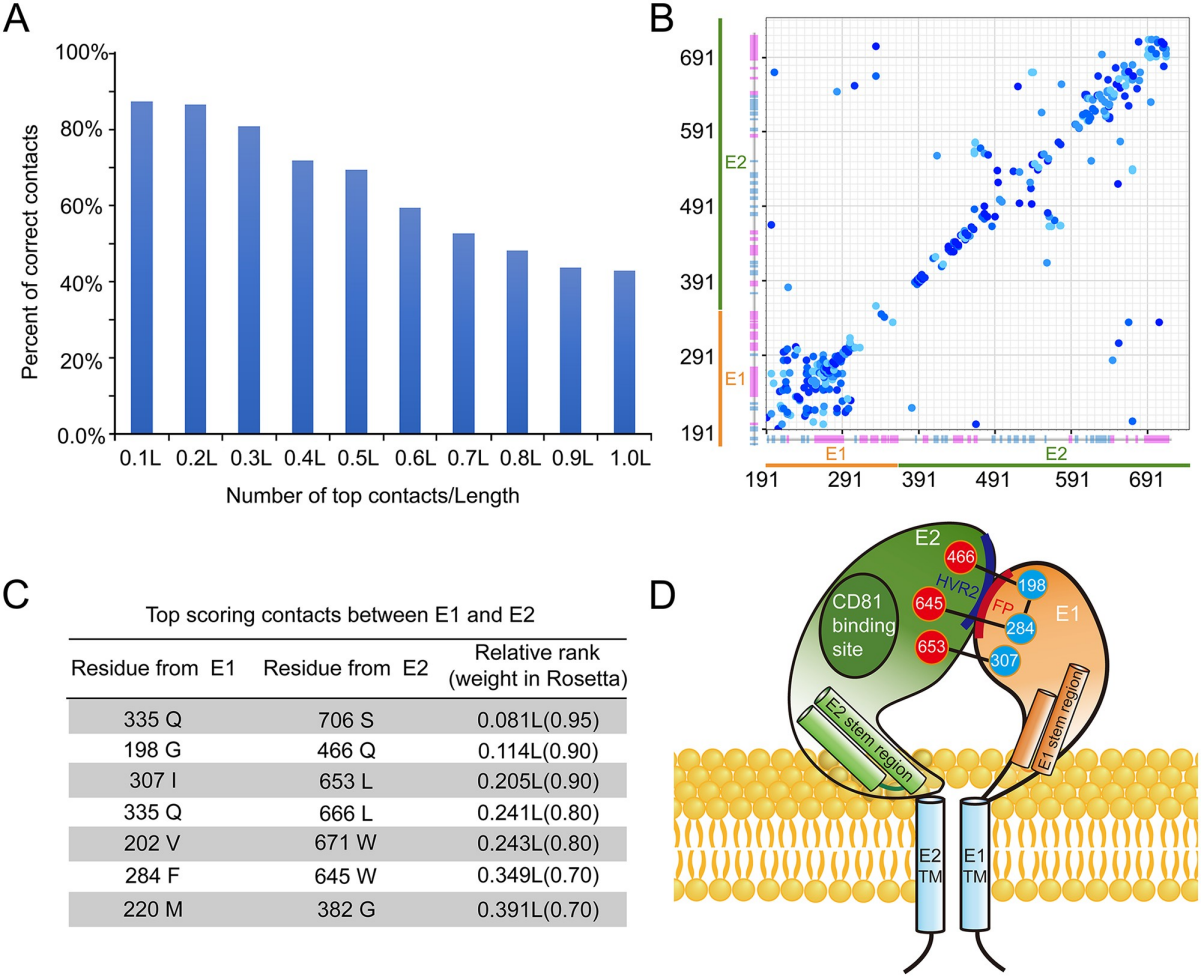
Coevolution based structural modeling of HCV E1E2 heterodimer. (A) Histogram of the accuracy of the top-ranked contacts. The accuracy of the predicated contacts is calibrated using the crystal structures of E2 core. (B) The top 0.5L contacts that are used in the Rosetta modeling protocol are shown as a contact map. Residue numbers of E1 and E2 are labeled and the predicated secondary structures of E1 and E2 are shown as bars with α-helix in magenta and β-sheet in cyan. (C) A table of the predicted top scoring contacts between E1 and E2. (D) A cartoon representation of E1E2 heterodimer on HCV envelope. The residues that may form contacts as well as the locations of the fusion peptide (FP), hyper variable region 2 (HVR2) and the CD81 binding site are shown in the model.

The accuracy of coevolution-based contact prediction depends on the availability of a large amount of diverse (< 90% sequence identity) sequences where coevolving residues can be detected unambiguously. There are about 50,000 different sequences of HCV glycoproteins in the database. Although these sequences are not diverse enough to guarantee accurate contact predictions based on the previous studies [54], we hypothesized that the massive amount of somewhat different (about 95% sequence identity) sequences may still contain valuable coevolution signals. We calibrated the prediction accuracy using residue pairs present in the crystal structures of E2 (PDB entry: 4MWF and 4WEB) [28, 29] (Fig 5A). The top 0.5L (L is the sum of the length of E1 and E2) predicted contacts are expected to contain 70% correct predictions, and the contacts between E1 and E2 among these predictions are listed in Fig 5C. The calibration also allowed us to assign a probability of being correct to each predicted contact, and the top 0.5L contacts that are separated by at least two residues (Fig 5B) are used as constraints for modeling with Rosetta.

In addition to the coevolution constraints, we used the partial structures of E1 (excluding the swapped beta hairpin from PDB entry: 4UOI) and E2 (PDB entry: 4MWF and 4WEB) as templates to model the complete ectodomains of E1 and E2 with Rosetta. The top five models ranked by Rosetta energy function (including atom pair constraints) are inspected and selected based on the agreement to the EM density and the satisfaction to the coevolution constraints between E1 and E2. The selected models of E1E2 heterodimer were manually docked into the EM density and further refined using Rosetta with coevolution constraints. The resulting model shows that the ectodomains of both E1 and E2 can be divided into two parts, an N-terminal region and a stem region (Fig 4B), and E1 and E2 have two interfaces: one locates at the membrane distal ends of the N-terminal regions and the other one stays near the membrane proximal ends of the stem regions (Figs 4 and 5D). The modeled N-terminal region of E2 is similar to the solved crystal structures, except that the hyper variable loop regions are rebuilt by the Rosetta loop modeling protocol. Deletion of hyper variable region 2 (HVR2) has been reported to abolish the formation of E1E2 heterodimers, indicating its important role in E1E2 heterodimer formation [56], which is consistent with our model where HVR2 comprises a large portion of the E1E2 interface (Fig 4B). Moreover, the model is also in agreement with the previously results showing that the back sheet region of E2 may interact with E1 [57]. In addition, a broadly neutralizing antibody AR3C has been showed to be able to block the binding of CD81 to HCV [28, 43]. The superimposition of the E2-AR3C complex structure [28] with the E1E2 heterodimer model shows that the CD81 binding site locates on the side of the E1E2 heterodimer, away from the E1E2 interface (S4C Fig).

The stem regions are commonly found in the glycoproteins in the *Flaviviridae* family [10, 58] and may play important roles in viral entry [59–63]. In the E1E2 structural model, the stems regions are composed of helices and interact with each other, which is in agreement with the strong coevolution signal between Q335 and S706 (Fig 5C). In addition, these helices are hydrophobic and have positive WWIHS scores [64], suggesting they may interact with the lipid membrane.

It is noteworthy that the putative fusion peptide of E1 (residue 272–285: CSAMYVGDLCGSVF), which has been suggested to be important for triggering the fusion process during HCV entry [15, 59, 65–68], forms a helix in our model and interacts directly with E2 (Fig 4B). This interaction is supported by the strong coevolutional signal between residues F284 of E1 and W645 of E2 (Fig 5C and 5D). Moreover, the structural modeling of E1 by Rosetta also gives several alternative conformations of the fusion peptide with comparable Rosetta energies. Among them, the extended helix of the peptide could bend in the middle and form a helical hairpin, suggesting that large conformational changes are allowed for the fusion peptide, which may be relevant to the fusion process. In addition, since the fusion peptide is quite hydrophobic, it could be unstable if exposed in the absence of E2, which may explain why E2 is required for the functional expression of E1.

In order to verify the model, we generated two deletion mutants at the interface between E1 and E2, including a HVR2 deletion mutant on E2 and a putative fusion peptide deletion mutant on E1. Moreover, two double mutants of the coevolving residues that may form hydrophobic contacts with each other at the E1E2 interface, including I307S &L653S and F284S&W645S, were also made. In parallel, another version of the two double mutants, I307R&L653R and F284R&W645R, were constructed to increase the probability of disrupting the interface. All the mutants were generated based on the E1E2-DHD15 construct and expressed in insect cells, which would only produce E1E2 heterodimers. However, none of the mutants were detected in supernatants, and the western blot data showed that all these mutants were expressed but retained intracellularly (S5A and S5B Fig), suggesting that the heterodimers were not formed properly probably due to the disruption of the interface between E1 and E2. By contrast, a single mutant Q466R, which showed strong coevolving signal with residue G198 on E1 (Fig 5C and 5D), could be expressed and secreted into supernatant (S5A and S5B Fig). Since Q466 locates at the peripheral region of the interface in the model (Fig 5D), therefore may not be able to affect the interface as other mutated hydrophobic residues, and indeed, the EM imaging of this mutant did not show any obvious difference with the wild type samples (S5C Fig).

### Interactions of E1E2 heterodimer with the cellular receptors

**Fig 6:**
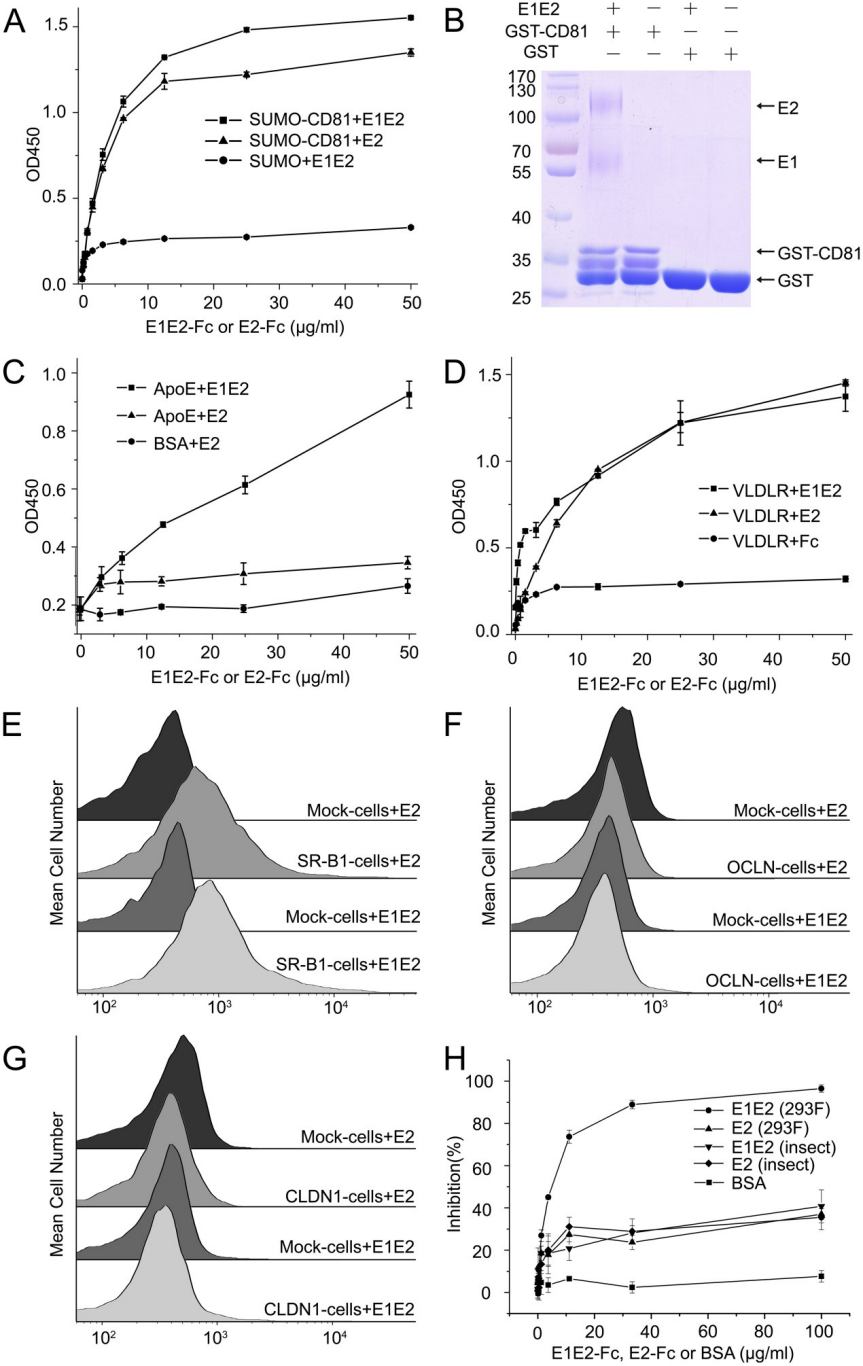
Interactions of HCV E1E2 heterodimer with the cellular receptors. (A) ELISA data show that both E1E2-Fc and E2-Fc bind to CD81. (B) GST pull-down assays show that E1E2-Fc binds to CD81. (C) ELISA data show that E1E2-Fc binds to ApoE, whereas E2-Fc has no obvious binding affinity to ApoE. (D) ELISA data show that E1E2-Fc and E2-Fc bind to VLDLR similarly. (E) FACS data show that both E1E2-Fc and E2-Fc bind to the SR-B1 transfected HEK293 cells. (F) Both E1E2-Fc and E2-Fc show no binding affinity to the OCLN transfected HEK293 cells. (G) Both E1E2-Fc and E2-Fc show no binding affinity to the CLDN1 transfected HEK293 cells. (H) Both E1E2-Fc and E2-Fc can block the infection of HCVcc. The ELISA data shown in (A), (C) and (D) are representative of three repeated experiments and presented as mean ± SD. The viral infection blocking data shown in (H) are representative of three repeated experiments and presented as mean ± SD.

A number of cell surface receptors have been reported for HCV cell entry, but the specific interactions between E1E2 and the receptors are not fully characterized. We first examined the interactions of E1E2 with the receptors using E1E2-Fc heterodimers. CD81 is a known HCV receptor that binds to E2 [8–11]. Indeed, the GST-pull down assays showed that the mammalian cell expressed E1E2-Fc heterodimer could bind to CD81 (Fig 6B), and according to the ELISA assays, the CD81 binding affinities of E1E2-Fc and E2-Fc were similar (Fig 6A), suggesting that CD81 might bind to E2 directly and may not have interactions with E1. This is consistent with the modeled structure of E1E2, where the CD81 binding site locates on the side of E2, far away from the E1E2 interface (Fig 5D and S4C Fig). By contrast, apolipoprotein E (ApoE) has been reported to facilitate the HCV entry through its interaction with E1 [16]. The ELISA results showed that E1E2-Fc could bind to ApoE, whereas E2-Fc only bound to ApoE at background level, confirming the recognition between ApoE and E1 (Fig 6C). Among the HCV receptors, very-low-density lipoprotein receptor (VLDLR) has been shown to mediate HCV entry independent of CD81, and E2 plays an important role in this process [14]. Indeed, the ELISA results showed that both E1E2-Fc and E2-Fc bound to VLDLR similarly (Fig 6D), which is consistent with previously studies. In addition, we also tested the interaction between oligomeric E1E2 and CD81, and the results showed that the E1E2 oligomer could also bind CD81 (S7D Fig), as has been reported previously [24].

In the meantime, we also tested the interactions of E1E2-Fc heterodimer with other HCV receptors, including SR-B1, OCLN and CLDN1. Since these receptors are multi-pass transmembrane proteins and difficult to isolate, we transfected HEK293 cells with the full-length receptors fused with GFP and monitored the binding of E1E2-Fc heterodimer by FACS. Among them, SR-B1 could bind to both E1E2-Fc and E2-Fc similarly (Fig 6E), suggesting that SR-B1 might interact mainly with E2, which is in agreement with the previous results [12]. By contrast, we were not able to detect any binding signals of E1E2-Fc on the OCLN or the CLDN1 transfected cells (Fig 6F and 6G), this is somewhat unexpected as these two molecules have been shown to be indispensable for HCV entry to murine cells [17, 18]. However, it has also been reported that other cofactors might be required for E1E2 to recognize OCLN and CLDN1 [69], therefore they may not interact directly with E1E2 during viral entry. In parallel, we also tested the binding of E1E2-DHD15 with CD81, SR-B1, OCLN and CLDN1, respectively, and similar results were obtained (S8 Fig).

To further confirm the functional activities of the expressed E1E2 heterodimer, we tested the inhibition of HCV infection with the expressed E1E2 heterodimer or E2 homodimer. The results showed that both E1E2 heterodimer and E2 homodimer could block the HCV infection, therefore validating the functionality of the E1E2 heterodimer (Fig 6H). Among them, the E1E2-Fc heterodimer expressed in mammalian cells appeared to block the infection better than other constructs, suggesting that both E1 and E2 as well as the glycosylation pattern of the envelope proteins may all affect the viral entry. Taken together, these results confirmed that the expressed soluble E1E2 heterodimer described above is functional and could be applied to explore the HCV entry mechanism and might also be a valuable target for developing prophylactic vaccines against HCV.

## Discussion

Although HCV was identified nearly thirty years ago [70], the structure and the life cycle of HCV have not been fully understood. Current publications suggest that HCV cell entry is a multistep process involving a number of receptors in a temporally and spatially ordered manner [1, 2], and the two envelope glycoproteins, E1 and E2, are the key players in the viral cell entry process. Probably due to the complex folding and maturation process of E1 and E2, the native form of E1E2 is difficult to isolate [33, 35, 36]. E1 and E2 have been shown to form a heterodimer through their transmembrane domains on viral surface, and the folding and maturation of E1 and E2 may depend on each other [20, 25, 26]. To mimic the native expression of E1E2 glycoproteins, we utilize either an IgG Fc region or a *de novo* designed heterodimeric tag to substitute the transmembrane domains of E1 and E2, resulting in E1E2 heterodimers similar to the native form of E1E2 on HCV particles, which has been validated by the binding of neutralizing antibodies that recognize conformational epitopes on both E1 and E2. Previous evidence has shown that the intracellular forms of E1 and E2 might be assembled as non-covalent heterodimers, whereas the virion-associated envelope glycoproteins could form covalent dimers or oligomers stabilized by disulfide bonds, and the disulfide bond-linked E1E2 complexes were in a conformation competent for cell entry [24]. Interestingly, the E1E2 proteins expressed by our strategy usually contain two species, E1E2 heterodimers and oligomers, and they all form inter-subunit disulfide bonds and could bind to the neutralizing antibodies and cellular receptors, which is consistent with the findings about the virion-associated E1E2 glycoproteins.

Previously, similar co-expression systems have been used successfully with either GNA enrichment method or fusing E2 with an Fc tag to facilitate purification [33, 71–74]. An advantage of the strategy described here is that the E1E2 heterodimers are expressed as water-soluble forms and secreted into media with reasonable yields, which makes purification and functional characterization much easier than isolating the heterodimers from membranes with detergents. The EM images also show that the purified E1E2 heterodimers are mono-dispersed and suitable for further structural studies.

*De novo* protein design has recently shown significant success in therapeutic drugs, new enzymes and biocatalysts, drug delivery tools and other applications [75]. Here we use a *de novo* designed helical bundle DHD15 to induce soluble heterodimer formation by replacing the transmembrane domains of E1 and E2. The DHD15 tag has several advantages: (1) The two N-termini of DHD15 are close to each other, which can be fused to the ectodomains of E1 and E2 without introducing extra geometric hindrance. The helical bundle of DHD15 also mimics the conformation of the transmembrane domains of native E1E2; (2) DHD15 can form stable heterodimer quickly and facilitate the folding and maturation of E1 and E2; (3) Since DHD15 forms a heterodimer, it could maximize the yield of E1E2 heterodimer during expression, because in the case of Fc tag, both E1E1 and E2E2 homodimers are also produced during expression; (4) The helical bundle of DHD15 is quite rigid containing only 75 amino acids with no disulfide bonds or glycosylation sites, making it suitable for crystallographic and EM studies. The application of this *de novo* designed heterodimeric tag for HCV E1E2 glycoproteins suggest that computational protein design could be a powerful tool to facilitate biological researches.

HCV glycoproteins are challenging targets to study with current structural approaches due to the conformational flexibility, glycosylation and folding requirements, and only partial structural information is available for E1 and E2 [27–29, 76]. Given the high sequence variability and the availability of a vast number of sequences, the E1E2 of HCV is a reasonable target for the *in silico* modeling using coevolution information derived from sequence alignments. In the context of the low-resolution EM reconstruction, the intact E1E2 ectodomain has been modeled by Rosetta using the coevolution information regarding the residue contacts. The E1E2 heterodimer roughly forms a doughnut-like conformation with two interfaces between E1 and E2 (Fig 4). Both E1 and E2 ectodomains exhibit elongated electron densities in the EM reconstruction and can be divided into an N-terminal region and a stem region. The putative fusion peptide on E1, the HVR2 and the back-sheet region on E2 are involved in forming the membrane distal interface between E1 and E2 in our model. This is in consistent with previous experimental and computational studies, for example, the HVR2 region on E2 has been shown to play an important role in E1E2 heterodimer formation [56], and the coevolution analysis shows the critical role of the back sheet region on E2 in the E1E2 interface. Moreover, a recent high-throughput mutagenesis study also emphasizes the importance of HVR2 and the back-sheet region in the heterodimer formation [77]. The stem regions of E1E2 include hydrophobic helices, which might be involved in forming the membrane proximal interface between E1 and E2, since the coevolution analysis shows strong residue coupling signals between the stem regions of E1 and E2 (Fig 5C).

Rosetta modeling has been used before for generating a computational model of E1E2 heterodimer [78], which shares some structural features with our model. During the modeling process, we combine Rosetta modeling with the coevolution analysis, which has been shown to be able to improve the accuracy of Rosetta predictions [79], and the structural information from the 3D EM model. Several mutants have also been made to test the structural model, especially the residues at the E1E2 interface with coevolution signals. The results show that most of the mutants cannot be secreted into media, suggesting that they might be critical for the formation of E1E2 heterodimer.

Several cell surface receptors have been reported to be involved in HCV cell entry, however, the direct binding profiles between E1E2 heterodimer and the receptors are still incomplete. The binding assays based on the expressed soluble E1E2 heterodimer suggest that CD81 interacts with E2, which is consistent with the published results as well as the modeled E1E2 structure. Similarly, two other receptors, SR-B1 and VLDLR, are also mainly interacting with E2. By contrast, ApoE could bind to the E1E2 heterodimer rather than the E2 ectodomain alone, suggesting that E1 might be involved in viral attachment through ApoE. Interestingly, no binding signals are detected for E1E2 heterodimer with OCLN or CLDN1, which have been shown to be functional at late stages of HCV entry. One possibility is that these two tight junction proteins might be involved in the endocytosis process without having direct interactions with E1E2 or other co-factors are required for the binding to E1E2 [69]. The HCV infection inhibition assays also show that the expressed E1E2 heterodimer could block the viral infection effectively. As exposed proteins on HCV surface, the E1E2 heterodimer is the target of immune system and the soluble E1E2 heterodimer obtained here would be a promising target for generating antibodies and facilitate the development of prophylactic vaccines against HCV.

## Materials and methods

### Protein expression and purification

The cDNA sequences encoding E1 and E2 of HCV genotype 1b, Con1 (Accession number AJ238799) and genotype 1a, H77 (Accession number AF009606) were synthesized. In order to co-express E1 (residues 192–354, for both Con1 and H77) and E2 (residues 384–717, for both Con1 and H77) glycoproteins in insect cells, the cDNA fragments of E1 and E2 excluding the transmembrane domains were sub-cloned into a pFastBac Dual vector (Invitrogen) (E1E2), then mouse IgG Fc homodimeric fragment with a Flag and a 6xHis tag at its C-termini was fused to the C-termini of E1 and E2, respectively (E1E2-Fc). Similarly, a *de novo* designed heterodimeric tag (DHD15), which contains a 6xHis tag and a Flag tag at its C-termini, was fused to the C-termini of E1 and E2, respectively (E1E2-DHD15). In parallel, both E1 and E2 fused with mouse IgG Fc with a 6xHis tag at the C-termini were also individually cloned to the pFastBac vector (E1-Fc and E2-Fc). Similar cDNA fragments, including E1E2, E1E2-Fc, E1E2-DHD15 as well as E1-Fc and E2-Fc were sub-cloned into pMlink co-expression vector [80] for transient expression in HEK293F cells (Invitrogen).

For antibody expression, sequences of IGH526 and HCV1 Fab fragments were obtained from PDB (4N0Y, 4DGV). The cDNA sequences were sub-cloned into pMlink co-expression vector [80] with 6xHis tag fused at the C-terminus of light chain for transient expression in HEK293F cells (Invitrogen).

For receptor binding assays, the ectodomain of human VLDLR (residues 28–797) (Han lab, Xiamen University) with a C-terminal 6xHis tag was cloned into pFastBac vector for expression in insect cells. The full-length human SR-B1 (Sino Biological), OCLN (Sino Biological), and CLDN1 (Sino Biological) fused with C-terminal GFP were also individually cloned into a pTT5 vector for transient expression in HEK293 cells.

For protein expression in insect cells, baculoviruses of the target proteins were generated following the Bac-to-Bac baculovirus expression protocol (Invitrogen), then High-5 cells (Invitrogen) were used for protein expression in ESF921 medium (Expression Systems). The supernatants were collected after 72~96 hours and buffer-exchanged with 50 mM Tris, 150 mM NaCl at pH 8.0 by dialysis, then applied to Ni-NTA affinity column (Qiagen) and Flag M2 affinity column (GeneScript) before loading onto a HiLoad Superdex 200 prep grade column (GE Healthcare) with Tris-NaCl buffer (50 mM Tris, 150 mM NaCl at pH 8.0) for further purification. The purified proteins were loaded onto SDS-PAGE for detection.

For protein expression in mammalian cells, target protein constructs were transiently expressed in HEK293F cells following the manufacturer’s protocol (Invitrogen) using PEI as transfection reagent. The transfected cells were cultured in Gibco FreeStyle-293 medium (Invitrogen) at 37°C for 6 days, then the supernatants were collected for purification using the similar buffers and conditions described above.

A fragment of human CD81 (Genewiz) (residues 122–202) fused with either the small ubiquitin-like modifier (SUMO) or Glutathione S-transferase (GST) were expressed in *E*. *coli* BL21(DE3) cells (Novagen) using expression vector pET28a or pGEX6p-1. The soluble SUMO-CD81 or GST-CD81 were purified from the supernatants of cell lysates by Ni-NTA affinity column (Qiagen) followed by SEC chromatography using a HiLoad Superdex 75 prep grade column (GE Healthcare) with Tris-NaCl buffer (50 mM Tris, 150 mM NaCl at pH 8.0). The human apolipoprotein E was purchased from Novoprotein.

### SDS-PAGE and Western blot assays

Purified Fc- and DHD15-tagged E1E2 or E2-Fc proteins were separated by SDS-PAGE (6% or 8%) and stained with coomassie brilliant blue R-250 (Aladdin). For western blot detection, both supernatants and cell pellets were run on SDS-PAGE (8%) for separation and transferred onto a polyvinylidene difluoride (PVDF) membrance (Invitrogen). The membrane was probed with mouse anti-His tag antibody (1:1000 dilution; Proteintech) or mouse anti-Flag M2 antibody (1:1000 dilution; Sigma) followed by the HRP-conjugated rabbit anti-mouse IgG secondary antibody (Proteintech). After washing three times with the buffer (25mM Tris, 150mM NaCl, pH 7.4, 0.05% Tween-20), the membrane was incubated with Diaminobenzidine (DAB, Sigma) for detection.

### Electron microscopy and 3D reconstruction

10 μl of purified HCV glycoprotein was apply to the glow-discharged EM carbon grids and stained with 0.75% (wt/vol) uranyl formate. Negatively stained EM grids were imaged on a Tecnai T12 microscope (FEI) operated at 120 kV. Images were recorded at a nominal magnification of 67,000x, using a 4k x 4k Eagle CCD camera, corresponding to a pixel size of 1.74 Å per pixel on the specimen. e2boxer.py program in EMAN2 suite was used to pick particles. e2refine2d.py of EMAN2 was used to generate 2D averaging classifications. The initial model was generated using the program e2initialmodel.py, and e2refine_easy.py of EMNA2 was used for refinement and reconstruction. The final resolution was estimated at 27Å based on the gold standard criterion.

### Coevolution analysis

The homologs of HCV E1 and E2 were identified from Refseq [81] and Uniref [82] databases and aligned using BLASTP [83]. A pair of E1 and E2 sequences from the same protein sequence were concatenated and the resulting alignment was filtered using HHfilter (-id 95 -cov 75) [84]. The filtered alignment was analyzed using GREMLIN [55] with two sets of parameters: (1) -e 0, -n 100, -w 0.8; and (2) -e 0, -n 100, -w 0.9. The resulting scores from the two CCMpred runs were averaged to obtain the final coevolution score of each pair of residues. All the predicted contacts were ranked by the strength of coevolution and extracted the top L (L is the length of E1 and E2) contacts. Some of the contacting residues from the top predictions were present in the crystal structures of E2 (PDB ids: 4MWF and 4WEB), therefore could be used to evaluate the accuracy of prediction. The prediction accuracy at each rank iL (L is the length of E1 and E2, and i = 0.1, 0.2, …, 1.0) were calculated as the number of correctly predicted contacts in the experimental structure divided by the total number of predicted contacting pairs that are present in the experimental structure. A predicted contacting residue pair is considered to be correct if the shortest distance between the residues in the structure is below 6 angstroms.

### Structural modeling

Both E1 and E2 ectodomains were partitioned into two parts, one N-terminal region (the first 125 residues for E1 and the first 280 residues for E2) and a highly hydrophobic region. Then RosettaCM [85] protocol was applied to model the N-terminal regions of E1 and E2, and used the partial crystal structures of E1 (PDB: 4UOI) and E2 (PDB: 4MWF and 4WEB) as templates. In addition, the top 0.5L predicted contacts were also used as constraints to aid the modeling. The constraints were set up as previously described [53], so that satisfying a contact was rewarded while missing a contact is still tolerated. The C-terminal regions were *de novo* modeled with coevolution-derived constraints.

E1 core region (E1c), E1 stem region (E1s), E2 core region (E2c), and E2 stem region (E2s) were modeled separately. The average pairwise TMscore (roughly means the percent of residues that can be aligned within 5 Å) of top 10 (out of thousands) models ranked by Rosetta energy function is a good estimator for modeling accuracy [53]. The accuracy of E1c, E1s, E2c, and E2s are 0.47, 0.41, 0.70, and 0.44 by TMscore, respectively. The models with the lowest Rosetta energy were selected except E1c. The E1c model has the second lowest energy as this model agrees better with the coevolving residues between E1 and E2. Guided by both the EM reconstruction and the coevolution constraints, we manually arranged E1c, E1s, E2c, and E2s together to generate a model of E1E2 heterodimer. The flexible loops in this model were removed first and then rebuilt and refined in the context of the whole structure using Rosetta hybridize protocol [85].

### Pull-down assays

Glutathione-Sepharose 4B beads (GE Healthcare) were mixed with GST-CD81 or GST protein alone (approximately 50 μg) in 100 μl PBS, then the beads were incubated with E2-Fc or E1E2-Fc (about 20 μg protein) in 800 μl PBS on a rotary shaker for 2 hrs at 4 °C. After washing 3 times with PBS, the beads were boiled and centrifuged before loading onto SDS-PAGE for detection with coomassie brilliant blue R250.

### ELISA experiments

The expressed receptors (VLDLR, SUMO-CD81), Fab fragments (IGH526 and HCV1), antibody AR3A and ApoE (Novoprotein) were coated onto 96-well MaxiSorp plates (Nunc) with ∼2 μg protein per well at 4 °C overnight. The plates were blocked with the TBST buffer (25mM Tris, 150mM NaCl, pH 7.4, 0.05% Tween-20) containing 5% (w/v) BSA for 3 hrs. The purified E1E2 (E1E2-DHD15, E1E2-Fc) or E2-Fc were serially diluted and added to each well in a binding buffer (25mM Tris, 150mM NaCl, pH 7.4, 0.05% Tween-20, 1% BSA). Then the plates were incubated at room temperature for 3 hrs (for VLDLR and SUMO-CD81) or at 4 °C overnight (for ApoE). After incubation, plates were washed with the TBST buffer for five times. For E1E2-DHD15 detection, mouse anti-FLAG M2 antibody (Sigma) were added to each well at 1:1000 dilution and incubated at room temperature for 1 hr, followed by washing with TBST for five times. After washing, HRP-conjugated rabbit anti-mouse IgG antibody (Proteintech) was added to each well at 1:1000 dilution and the plates were incubated at room temperature for 1 hr. After washing five times with the TBST buffer, 100 μl of chromogenic substrate (1 μg/mL tetramethylbenzidine, 0.006% H_2_O_2_ in 0.05 M phosphate citrate buffer, pH 5.0) was added to each well and incubated for 30 min at 37°C. Then, 50 μl H_2_SO_4_ (2.0 M) was added to each well to stop the reactions. The plates were read at 450 nm on a Synergy Neo machine (BioTek Instruments).

### Flow cytometry

HEK293 cells were transfected with pTT5 vectors containing GFP tagged human SR-B1, OCLN or CLDN1. After 48 hrs of transfection, E1E2-DHD15, E1E2-Fc or E2-Fc (~20 μg) were added to the transfected cells in PBS and incubated on a rotary shaker for 2 hrs at room temperature and followed by washing three times with PBS. For E1E2-DHD15 detection, mouse anti-Flag M2 antibody (1:1000 dilution; Sigma) was added and incubated on a rotary shaker for 1 hr, followed by washing three times with PBS. After washing, anti-mouse IgG, F(ab')_2_ fragment Alexa Fluor 647 Conjugate antibody (1:1000 dilution; Cell Signaling Technology) was added to the cells and incubated on a rotary shaker for 1 hr at room temperature. After washing three times with PBS, cells were analyzed by a LSR Fortessa flow cytometer (Becton Dickinson). Data analysis was performed using FlowJo software (Tree Star).

### Deglycosylation assays

Either insect or mammalian cell expressed E1E2-DHD15 (~10 μg) were incubated with endo-β-N-acetylglucosaminidase H (Endo H) (50 U) at 37 °C for 1 hr according to the manufacturer’s instruction (Novoprotein). Then the treated proteins were load onto SDS-PAGE for detection. The treated proteins were also purified by SEC and then loaded onto EM grids for negative staining and imaging.

### Viral infection blocking assays

To perform an HCV infection-blocking assay, Huh-7 cells (Stem Cell Bank, Chinese Academy of Sciences) seeded at 1 x 10^4^ cells/well in a 96-well plate were incubated with serially diluted E1E2-Fc (insect cell expressed), E2-Fc (insect cell expressed), E1E2-Fc (HEK293 expressed), E2-Fc (HEK293 expressed) or bovine serum albumin (BSA) (New England BioLabs). After 1 hr incubation at room temperature, about 100 focus-forming units of JFH1 HCV cell culture (HCVcc) were added to the cells, and the protein-virus mixture was removed after 6 hrs of infection. After 3 days of cell culture in complete DMEM, cells were fixed with 2% paraformaldehyde and blocked with buffer (3% BSA, 0.3% Triton X-100, and 10% FBS in PBS), followed by incubation with anti-HCV NS5A MAb (Abmart), Alexa Fluor 488-conjugated donkey anti-mouse IgG and Hoechst dye. The infection efficiency was determined by counting the number of NS5A-positive fluorescent foci under a fluorescence microscope.

## Supporting information

S1 Fig Expression of HCV E2-Fc and E1-Fc in insect cells. (A) The SEC profile and the SDS-PAGE of the purified E2-Fc expressed in insect cells. The SEC peak of E2-Fc homodimer is indicated by a black arrow. (B) A negative staining EM image of E2-Fc particles (left; red circles; bar, 100 nm) and the representative 2D averaging classes (right; bar, 10 nm). (C) Western blot assay detecting E1 in supernatant (S) and cell pellet (P) of the E1-Fc expressed in insect cells.
(TIF)

S2 Fig Expression of HCV E2-Fc, E1-Fc and E1E2-Fc in HEK293 cells. (A) The SEC profile and the SDS-PAGE of the purified E2-Fc expressed in HEK293 cells. The SEC peak of E2-Fc homodimer is indicated by a black arrow. (B) A negative staining EM image of E2-Fc particles (left; red circles; bar, 100 nm) and the representative 2D averaging classes (right; bar, 10 nm). (C) Western blot assay detecting E1 in supernatant (S) and cell pellet (P) of the E1-Fc expressed in HEK293 cells. (D) SDS-PAGE of E1E2-Fc from HCV genotype 1a H77 expressed in HEK293 cells under reducing condition.
(TIF)

S3 Fig Negative staining EM images of the insect cell expressed E1E2-Fc and E1E2-DHD15 oligomers. (A) A negative staining EM image showing the oligomeric E1E2-Fc particles (left; red circles; bar, 100 nm). The representative 2D averaging classes are also shown (right; bar, 10 nm). (B) A negative staining EM image showing the oligomeric E1E2-DHD15 particles (left; red circles; bar, 100 nm). The representative 2D averaging classes are also shown (right; bar, 10 nm).
(TIF)

S4 Fig Negative staining EM of E1E2-DHD15. (A) A negative staining EM image of E1E2-DHD15 particles in acidic condition (pH 5.5) (left; red circles; bar, 100 nm) and the representative 2D averaging classes (right; bar, 10 nm). (B) Three views of the 3D EM reconstruction (gray) of E1E2-DHD15 in acidic condition. (C) Superposition of the crystal structure of HCV E2 core in complex with the neutralizing antibody AR3C (cyan) with the E1E2-DHD15 structural model shows that the CD81 binding site on E2 (green) is overlapped with the AR3C binding site, away from the E1E2 interface.
(TIF)

S5 Fig Mutagenesis analysis of E1E2 heterodimer. (A) Western blot assay detecting E1 of the E1E2-DHD15 mutants in supernatants (S) and cell pellets (P). (B) Western blot assay detecting E2 of the E1E2-DHD15 mutants in supernatants (S) and cell pellets (P). (C) A negative staining EM image showing the particles of the E1E2-DHD15 mutant (Q466R) (left; red circles; bar, 100 nm). The representative 2D averaging classes are also shown (right; bar, 10 nm).
(TIF)

S6 Fig Deglycosylation analysis of E1E2-DHD15 heterodimers. (A) SDS-PAGE of the insect cell expressed E1E2-DHD15 treated with or without Endo H. (B) SDS-PAGE of the HEK293 cell expressed E1E2-DHD15 treated with or without Endo H. (C) A negative staining EM image showing the insect cell expressed E1E2-DHD15 particles after Endo H treatment (left; red circles; bar, 100 nm). The representative 2D averaging classes are also shown (right; bar, 10 nm).
(TIF)

S7 Fig Interactions of E1E2-Fc heterodimer and oligomer expressed in HEK293 cells with neutralizing antibodies and CD81. (A)-(D) ELISA data show that both E1E2-Fc heterodimer and oligomer expressed in HEK293 cells can bind to neutralizing antibodies AR3A, IGH526 and HCV1 as well as CD81. The ELISA data shown in (A)-(D) are representative of three repeated experiments and presented as mean ± SD.
(TIF)

S8 Fig Interactions of E1E2-DHD15 with the cellular receptors. (A) FACS data show that E1E2-DHD15 binds to the CD81 or the SR-B1 transfected HEK293 cells. (B) FACS data show that E1E2-DHD15 has no binding to the CLDN1 or the OCLN transfected HEK293 cells. (C) ELISA data show that both insect and mammalian cell expressed E1E2-DHD15 can bind to CD81. The ELISA data shown in (C) are representative of three repeated experiments and presented as mean ± SD.
(TIF)

S9 Fig SDS-PAGE of the purified Fab fragments of neutralizing antibody IGH526 and HCV1 under reducing and non-reducing conditions. (TIF)
