# Supplementary figures and images for: Functional expression and characterization of the envelope glycoprotein E1E2 heterodimer of hepatitis C virus
Source: PLoS Pathog. 2019 May 22;15(5):e1007759. doi: 10.1371/journal.ppat.1007759 (PMC6530877; doi:10.1371/journal.ppat.1007759)

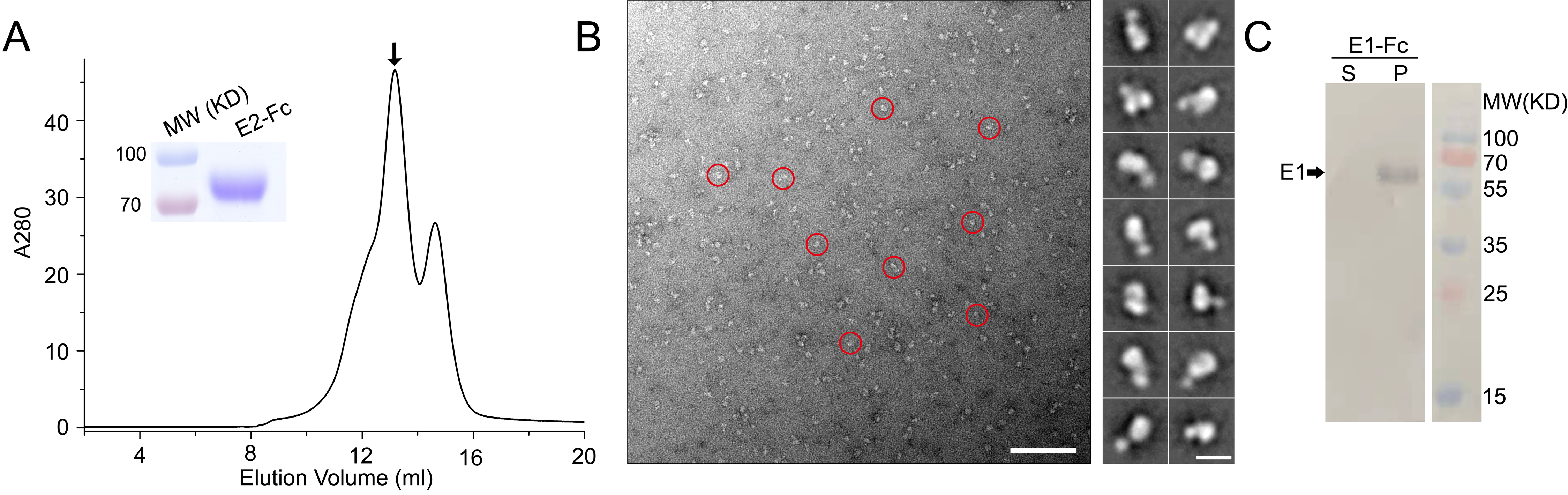

Supplement: S1 Fig — (A) The SEC profile and the SDS-PAGE of the purified E2-Fc expressed in insect cells. The SEC peak of E2-Fc homodimer is indicated by a black arrow. (B) A negative staining EM image of E2-Fc particles (left; red circles; bar, 100 nm) and the representative 2D averaging classes (right; bar, 10 nm). (C) Western blot assay detecting E1 in supernatant (S) and cell pellet (P) of the E1-Fc expressed in insect cells. (TIF) [file ppat.1007759.s001.tif]

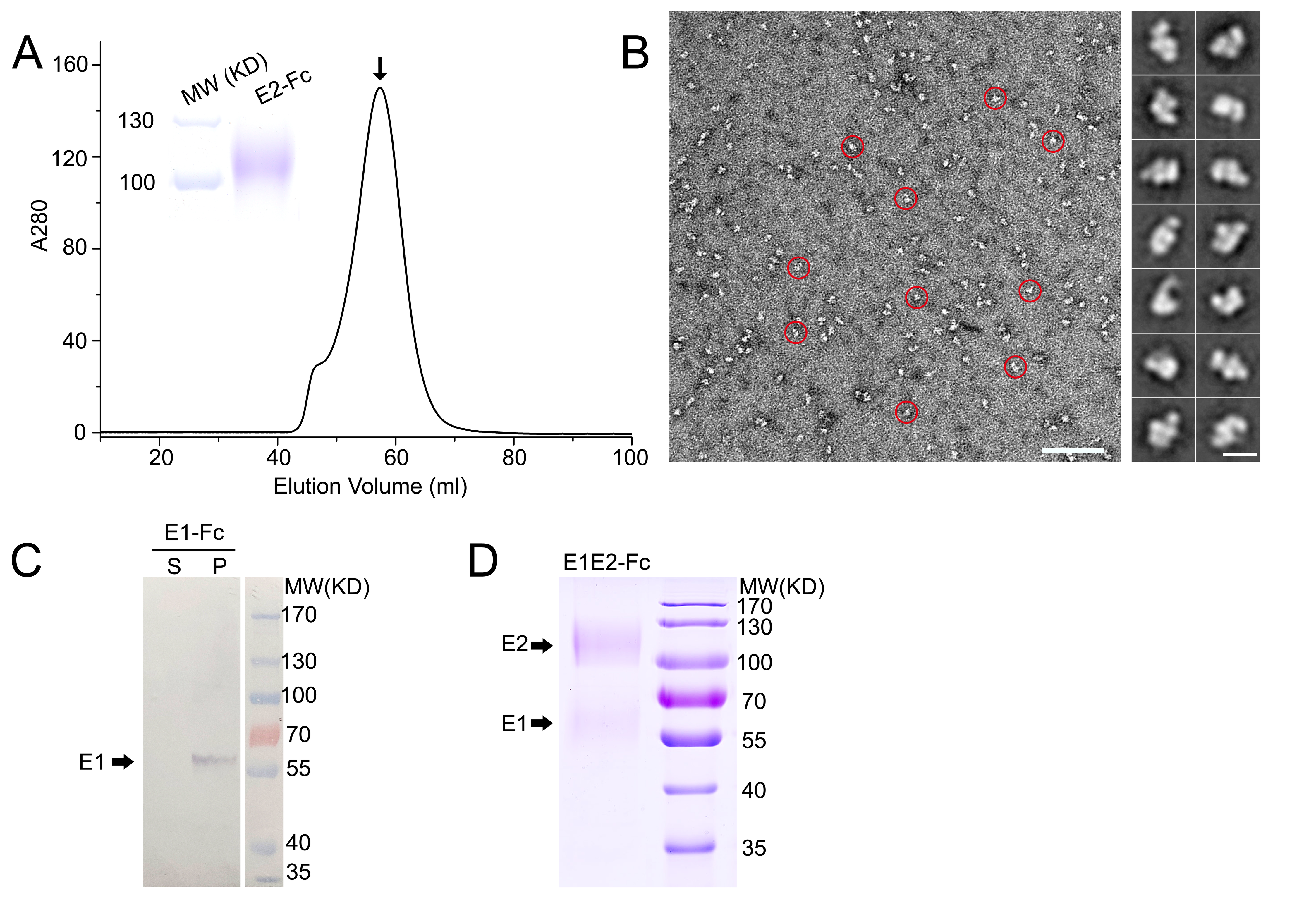

Supplement: S2 Fig — (A) The SEC profile and the SDS-PAGE of the purified E2-Fc expressed in HEK293 cells. The SEC peak of E2-Fc homodimer is indicated by a black arrow. (B) A negative staining EM image of E2-Fc particles (left; red circles; bar, 100 nm) and the representative 2D averaging classes (right; bar, 10 nm). (C) Western blot assay detecting E1 in supernatant (S) and cell pellet (P) of the E1-Fc expressed in HEK293 cells. (D) SDS-PAGE of E1E2-Fc from HCV genotype 1a H77 expressed in HEK293 cells under reducing condition. (TIF) [file ppat.1007759.s002.tif]

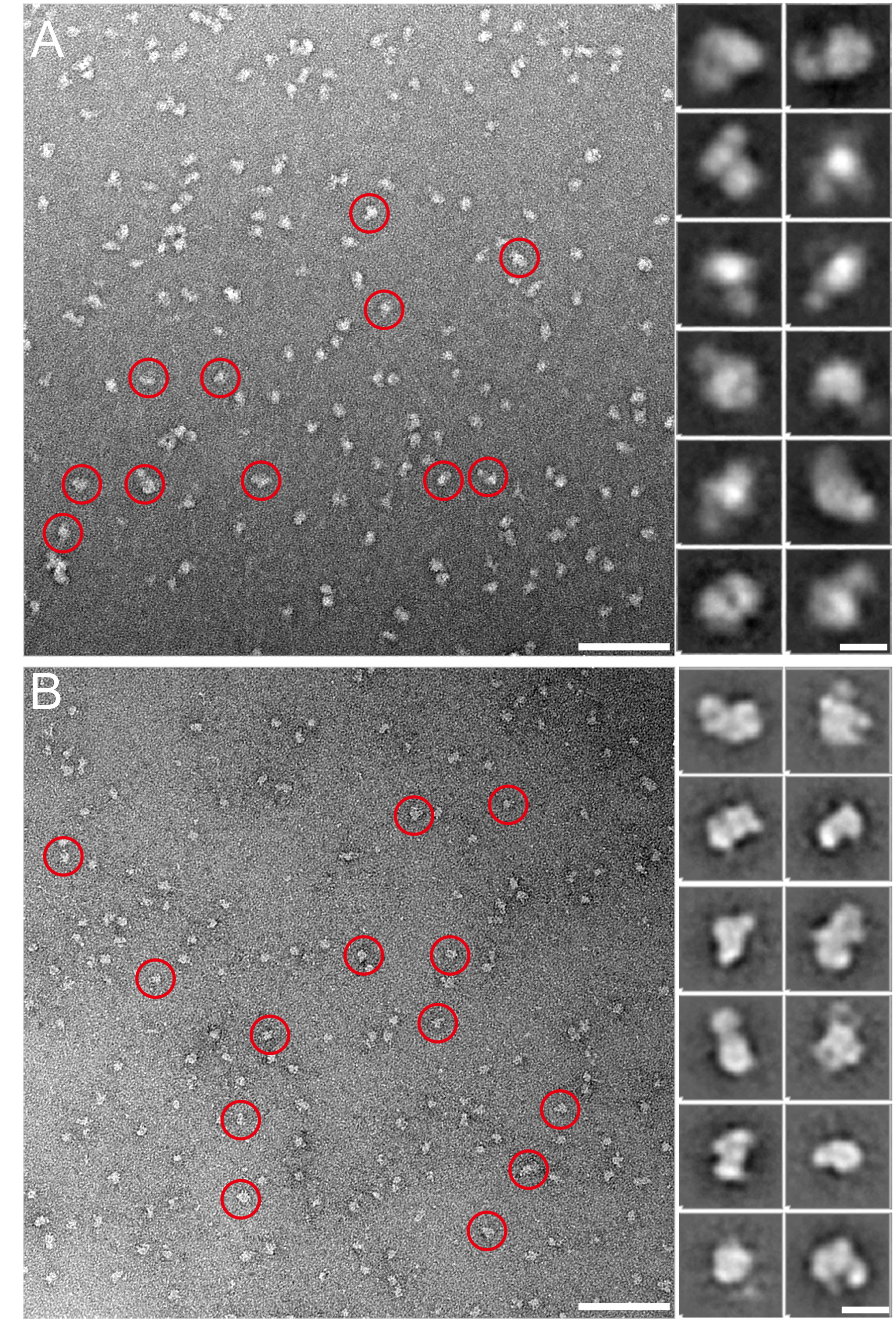

Supplement: S3 Fig — (A) A negative staining EM image showing the oligomeric E1E2-Fc particles (left; red circles; bar, 100 nm). The representative 2D averaging classes are also shown (right; bar, 10 nm). (B) A negative staining EM image showing the oligomeric E1E2-DHD15 particles (left; red circles; bar, 100 nm). The representative 2D averaging classes are also shown (right; bar, 10 nm). (TIF) [file ppat.1007759.s003.tif]

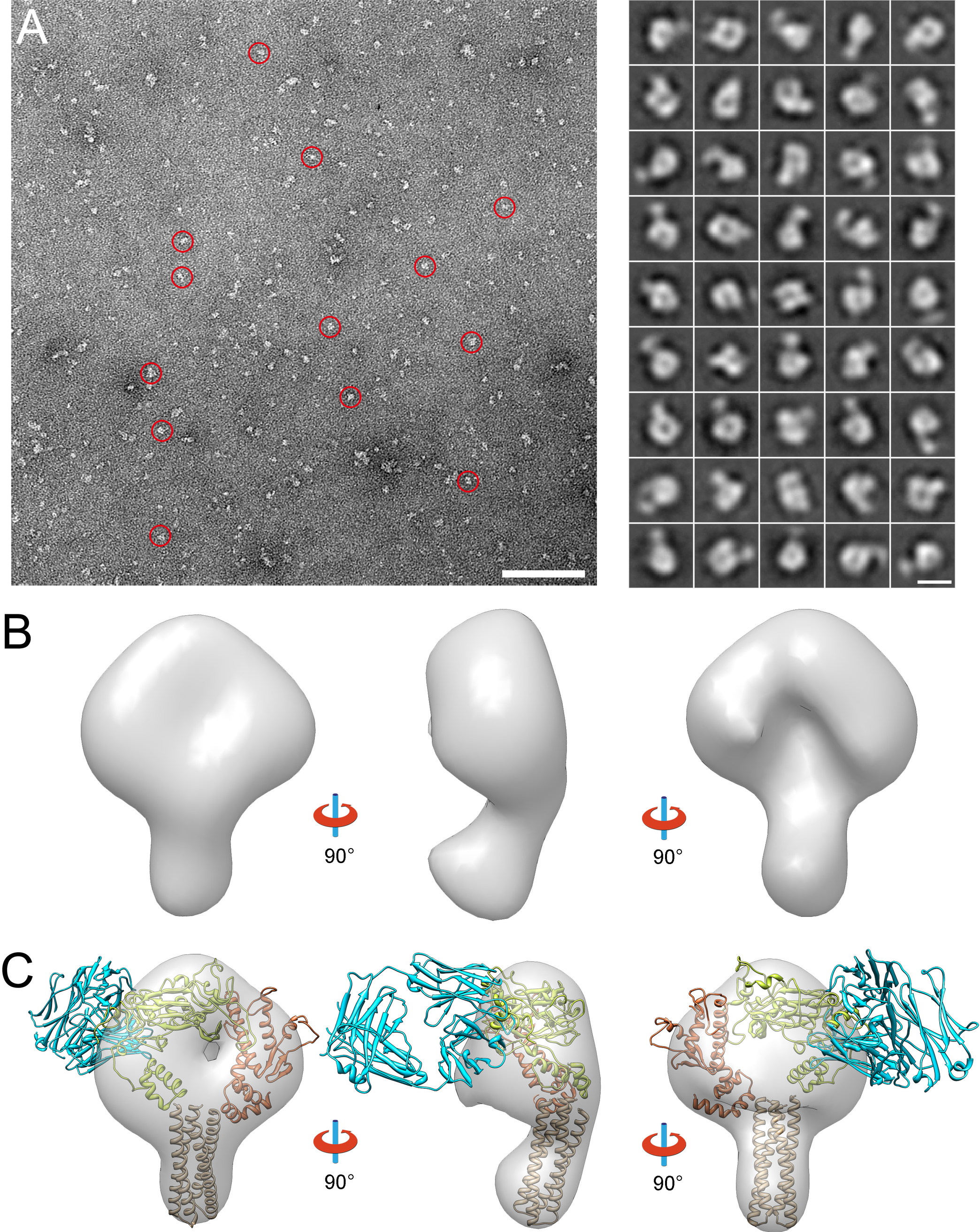

Supplement: S4 Fig — (A) A negative staining EM image of E1E2-DHD15 particles in acidic condition (pH 5.5) (left; red circles; bar, 100 nm) and the representative 2D averaging classes (right; bar, 10 nm). (B) Three views of the 3D EM reconstruction (gray) of E1E2-DHD15 in acidic condition. (C) Superposition of the crystal structure of HCV E2 core in complex with the neutralizing antibody AR3C (cyan) with the E1E2-DHD15 structural model shows that the CD81 binding site on E2 (green) is overlapped with the AR3C binding site, away from the E1E2 interface. (TIF) [file ppat.1007759.s004.tif]

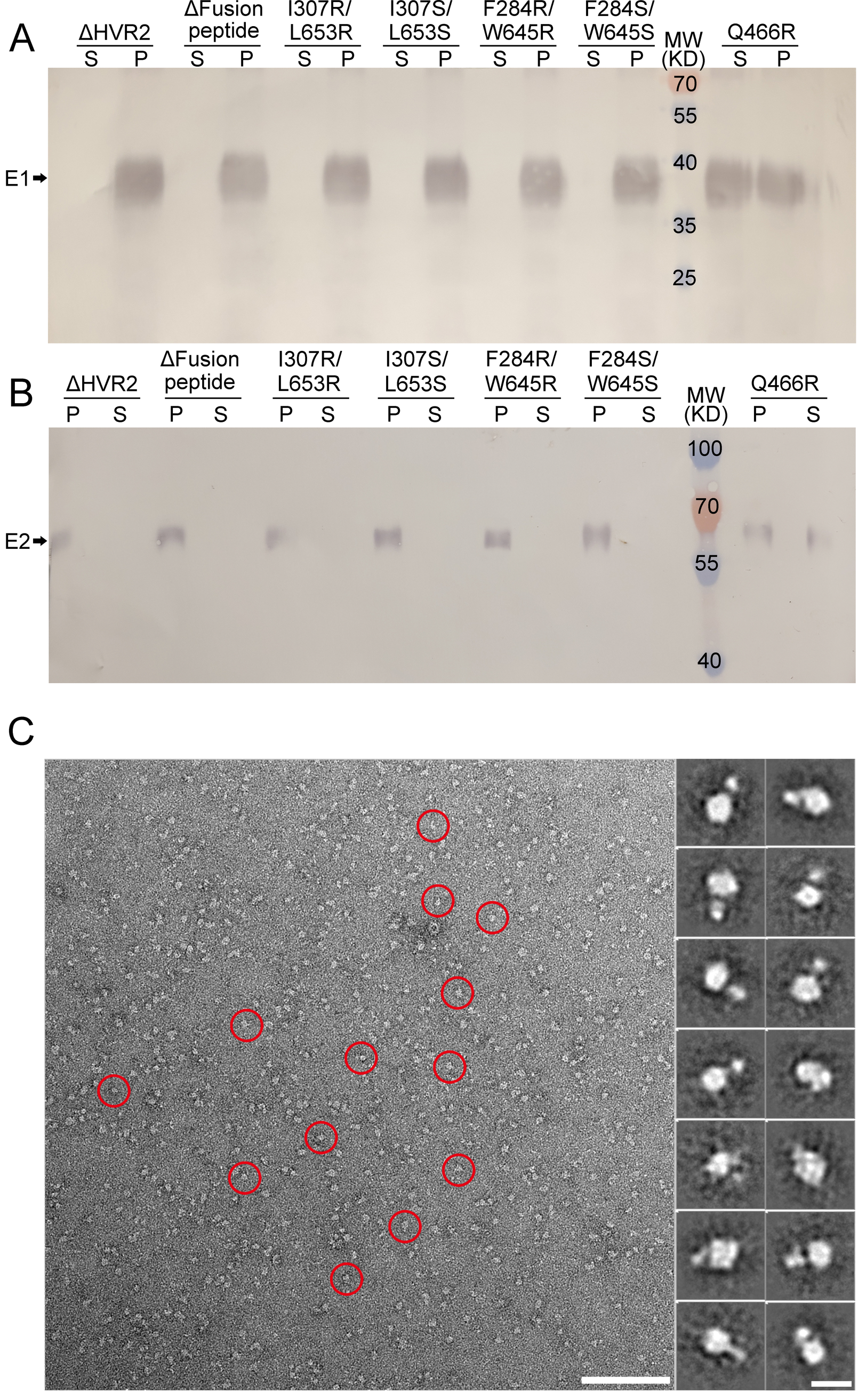

Supplement: S5 Fig — (A) Western blot assay detecting E1 of the E1E2-DHD15 mutants in supernatants (S) and cell pellets (P). (B) Western blot assay detecting E2 of the E1E2-DHD15 mutants in supernatants (S) and cell pellets (P). (C) A negative staining EM image showing the particles of the E1E2-DHD15 mutant (Q466R) (left; red circles; bar, 100 nm). The representative 2D averaging classes are also shown (right; bar, 10 nm). (TIF) [file ppat.1007759.s005.tif]

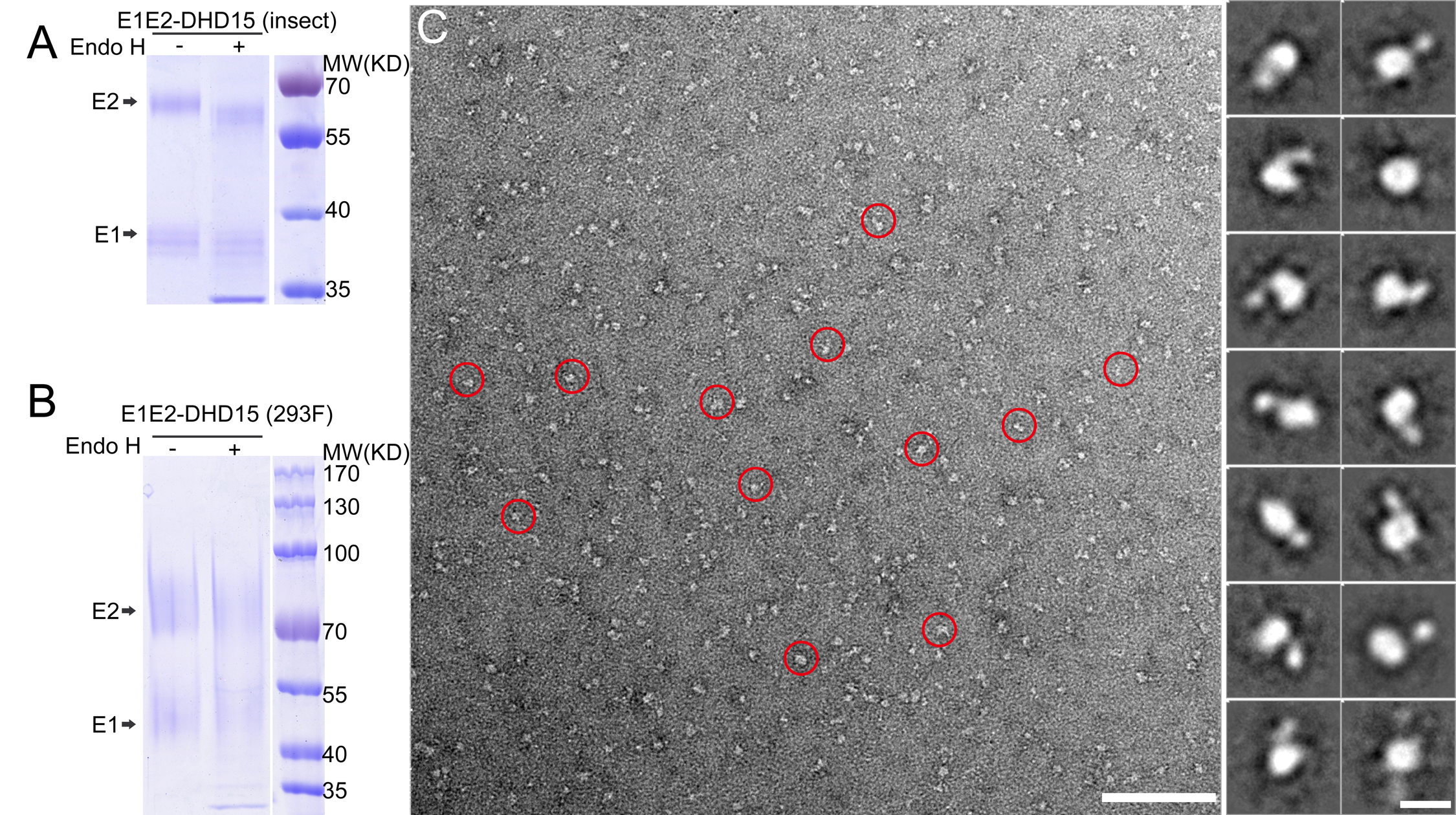

Supplement: S6 Fig — (A) SDS-PAGE of the insect cell expressed E1E2-DHD15 treated with or without Endo H. (B) SDS-PAGE of the HEK293 cell expressed E1E2-DHD15 treated with or without Endo H. (C) A negative staining EM image showing the insect cell expressed E1E2-DHD15 particles after Endo H treatment (left; red circles; bar, 100 nm). The representative 2D averaging classes are also shown (right; bar, 10 nm). (TIF) [file ppat.1007759.s006.tif]

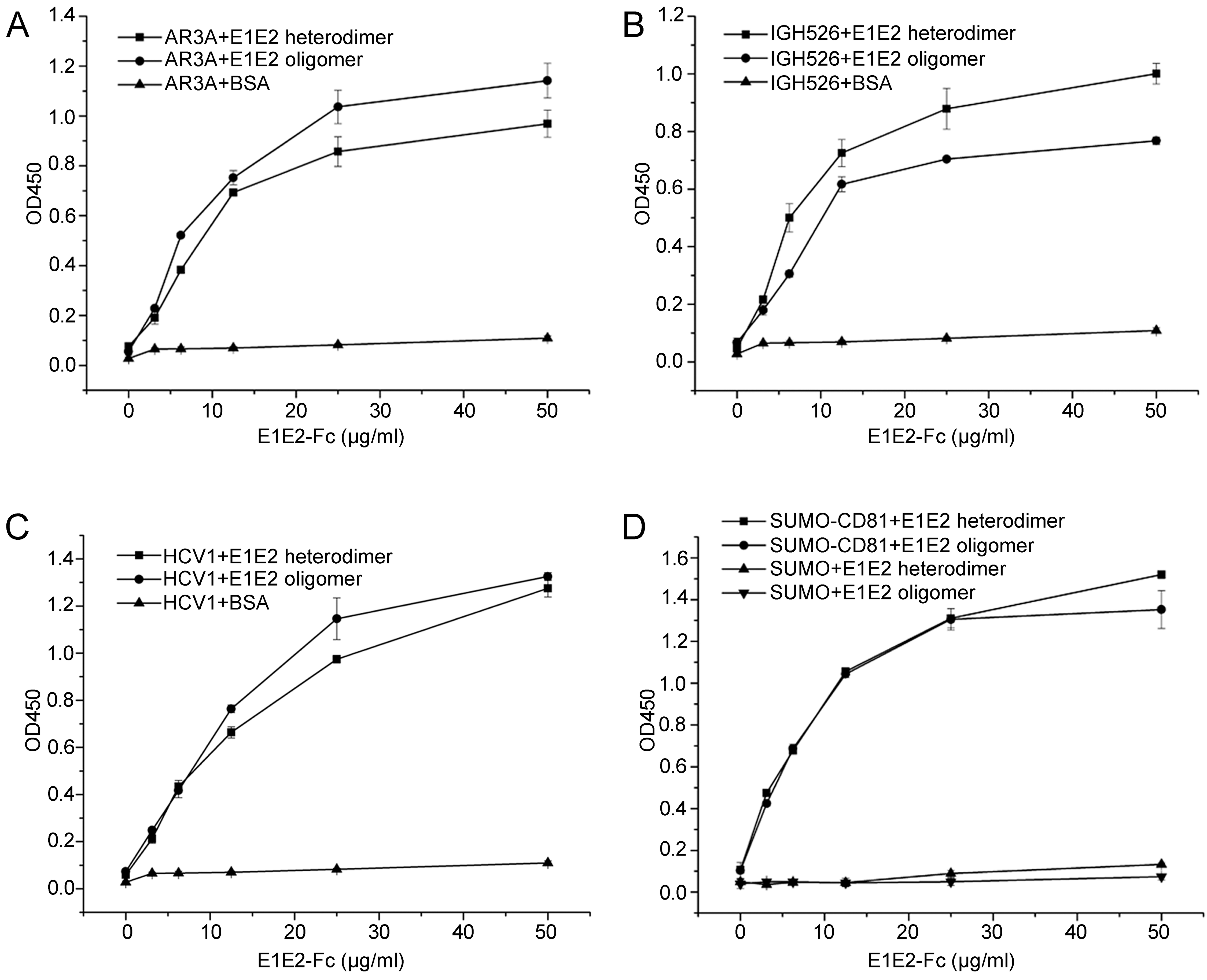

Supplement: S7 Fig — (A)-(D) ELISA data show that both E1E2-Fc heterodimer and oligomer expressed in HEK293 cells can bind to neutralizing antibodies AR3A, IGH526 and HCV1 as well as CD81. The ELISA data shown in (A)-(D) are representative of three repeated experiments and presented as mean ± SD. (TIF) [file ppat.1007759.s007.tif]

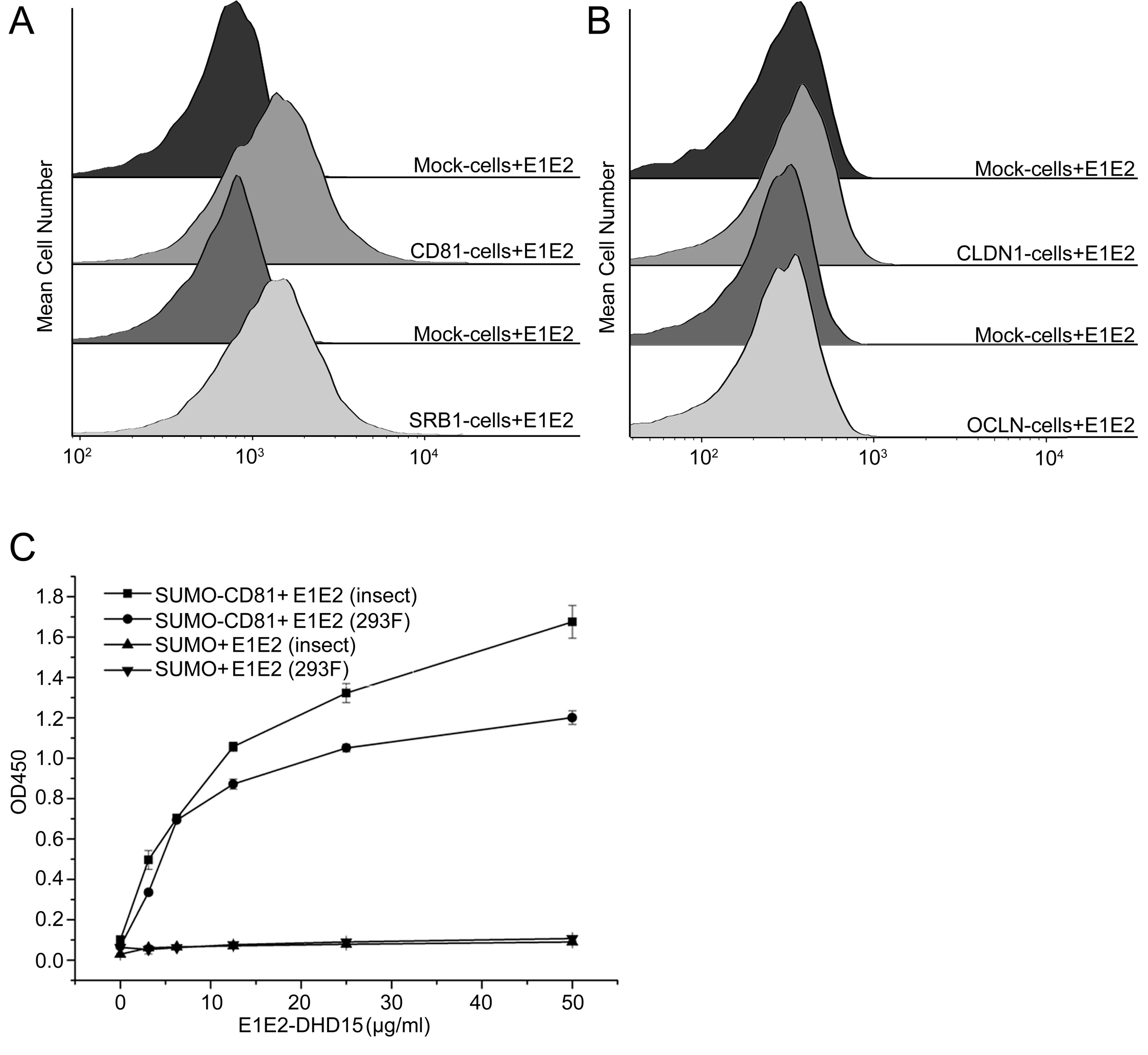

Supplement: S8 Fig — (A) FACS data show that E1E2-DHD15 binds to the CD81 or the SR-B1 transfected HEK293 cells. (B) FACS data show that E1E2-DHD15 has no binding to the CLDN1 or the OCLN transfected HEK293 cells. (C) ELISA data show that both insect and mammalian cell expressed E1E2-DHD15 can bind to CD81. The ELISA data shown in (C) are representative of three repeated experiments and presented as mean ± SD. (TIF) [file ppat.1007759.s008.tif]

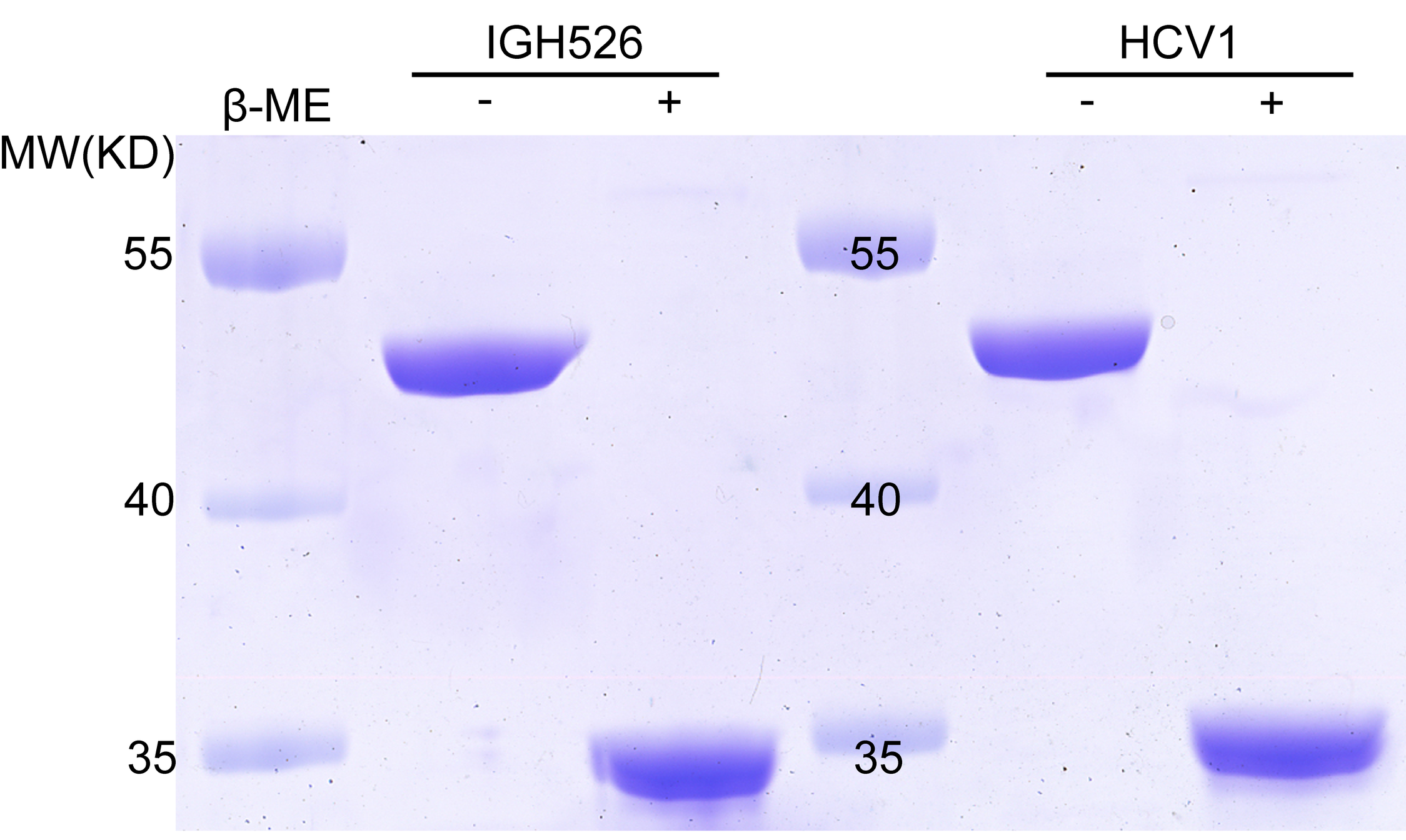

Supplement: S9 Fig — (TIF) [file ppat.1007759.s009.tif]
